# Temporal Dynamics of Natural Static Emotional Facial Expressions Decoding: A Study Using Event- and Eye Fixation-Related Potentials

**DOI:** 10.3389/fpsyg.2018.01190

**Published:** 2018-07-12

**Authors:** Anne Guérin-Dugué, Raphaëlle N. Roy, Emmanuelle Kristensen, Bertrand Rivet, Laurent Vercueil, Anna Tcherkassof

**Affiliations:** ^1^GIPSA-lab, Institute of Engineering, Université Grenoble Alpes, Centre National de la Recherche Scientifique, Grenoble INP, Grenoble, France; ^2^Department of Conception and Control of Aeronautical and Spatial Vehicles, Institut Supérieur de l’Aéronautique et de l’Espace, Université Fédérale de Toulouse, Toulouse, France; ^3^Laboratoire InterUniversitaire de Psychologie – Personnalité, Cognition, Changement Social, Université Grenoble Alpes, Université Savoie Mont Blanc, Grenoble, France; ^4^Exploration Fonctionnelle du Système Nerveux, Pôle Psychiatrie, Neurologie et Rééducation Neurologique, CHU Grenoble Alpes, Grenoble, France; ^5^Université Grenoble Alpes, Inserm, CHU Grenoble Alpes, Grenoble Institut des Neurosciences, Grenoble, France

**Keywords:** emotional facial expression, natural faces, event-related potential, eye fixation-related potential, temporal dynamics, General Linear Model

## Abstract

This study aims at examining the precise temporal dynamics of the emotional facial decoding as it unfolds in the brain, according to the emotions displayed. To characterize this processing as it occurs in ecological settings, we focused on unconstrained visual explorations of natural emotional faces (i.e., free eye movements). The General Linear Model (GLM; Smith and Kutas, 2015a,b; Kristensen et al., 2017a) enables such a depiction. It allows deconvolving adjacent overlapping responses of the eye fixation-related potentials (EFRPs) elicited by the subsequent fixations and the event-related potentials (ERPs) elicited at the stimuli onset. Nineteen participants were displayed with spontaneous static facial expressions of emotions (Neutral, Disgust, Surprise, and Happiness) from the DynEmo database (Tcherkassof et al., 2013). Behavioral results on participants’ eye movements show that the usual diagnostic features in emotional decoding (eyes for negative facial displays and mouth for positive ones) are consistent with the literature. The impact of emotional category on both the ERPs and the EFRPs elicited by the free exploration of the emotional faces is observed upon the temporal dynamics of the emotional facial expression processing. Regarding the ERP at stimulus onset, there is a significant emotion-dependent modulation of the P2–P3 complex and LPP components’ amplitude at the left frontal site for the ERPs computed by averaging. Yet, the GLM reveals the impact of subsequent fixations on the ERPs time-locked on stimulus onset. Results are also in line with the valence hypothesis. The observed differences between the two estimation methods (Average vs. GLM) suggest the predominance of the right hemisphere at the stimulus onset and the implication of the left hemisphere in the processing of the information encoded by subsequent fixations. Concerning the first EFRP, the Lambda response and the P2 component are modulated by the emotion of surprise compared to the neutral emotion, suggesting an impact of high-level factors, in parieto-occipital sites. Moreover, no difference is observed on the second and subsequent EFRP. Taken together, the results stress the significant gain obtained in analyzing the EFRPs using the GLM method and pave the way toward efficient ecological emotional dynamic stimuli analyses.

## Introduction

The investigation of the electrocerebral responses to emotional facial expressions (EFEs) is a privileged mean to understand how people process the emotions they see in others’ faces (Ahern and Scharwtz, 1985). To evaluate brain responses to EFE processing, most studies use the same experimental protocol. Pictures of EFE are presented during a short time and participants are asked to fixate at the center point of the image while the electroencephalographic signals are recorded. The brain response at the EFE presentation is estimated by averaging the EEG signal time-locked at this stimulus onset. Only synchronous activities elicited at the stimulus presentation contribute to this evoked potential (event-related potential, ERP) when averaging. A main assumption underlies this methodology, that of a unique potential elicited by the event of interest. If the presentation duration is very short and if there is only one ocular fixation at the image center and no eye movement afterward, this estimation for the evoked potential at the image onset is a good solution. Research based on this protocol shows two main stages in the time course of EFE processing. The first stage is a perceptual processing occurring early and stemming from the activity of occipital and temporal regions. The second stage is a conscious recognition one involving a more complex set of activations from frontal and subcortical structures (Adolphs, 2002). Some researchers have posited that the first stage is not impacted by valence and would merely reflect raw structural processing. This view is supported by some ERPs based studies on EFE processing. For instance, Eimer et al. (2003) found that emotional faces elicited higher amplitudes than neutral ones for late components but not for early ones. In the same vein, Almeida et al. (2016) found that arousal, but not valence of the EFE, modulates the amplitude of the N170. However, much more studies have shown that valence does in fact impact early EFE processing, unveiling a very rapid and early top-down modulation during this perceptual stage or at least “rapid emotion processing based on crude visual cues in faces” (Vuillemier and Pourtois, 2007). Indeed, differences in latency and amplitudes of ERP components can occur as early as the first 100 ms post-stimulation, e.g., P1 component (Batty and Taylor, 2003; Neath and Itier, 2015; Itier and Neath-Tavares, 2017), as well as modulations of both latency and amplitude of the face-specific N170 component at posterior temporal-occipital sites (Pizzagalli et al., 1999; Campanella et al., 2002; Blau et al., 2007; Hinojosa et al., 2015; Itier and Neath-Tavares, 2017) and of an anterior negative component around 230 ms (Balconi and Pozzoli, 2003). Moreover, a valence-dependent modulation of a component called early posterior negativity (EPN) between 150 and 300 ms at occipito-parietal sites has been found with a higher amplitude for EFEs than for neutral faces (Recio et al., 2011; Neath-Tavares and Itier, 2016; Itier and Neath-Tavares, 2017). This component can be computed by subtracting the ERP elicited by neutral faces to that of the emotional ones. If no subtraction is performed, the component is akin to a P2 component at posterior sites and a N2 one at anterior sites. In this article, the subsequent occurrence of a P2 and a P3 components at posterior sites will be referred to as a P2–P3 complex in order to avoid any confusion. Additionally, a modulation of late ERP components has been consistently reported and would reflect a conscious recognition process of EFEs. Hence, a valence-dependent amplitude modulation of a positive component around 350 ms at fronto-central sites and of the late positive potential (LPP) at all sites has been reported (Krolak-Salmon et al., 2001; Batty and Taylor, 2003; Trautmann-Lengsfeld et al., 2013). Moreover, Recio et al. (2011) reported an emotion-dependent modulation for EFE processing of a component akin to the LPP, called the late positive complex (LPC). This long lasting positivity component peaks at 500 ms over centro-parietal sites and is computed by subtracting the neutral ERP from the emotional ones. All in all, the time-course of EFE processing is now precisely documented by studies using the same experimental protocol. This said, the question remains as to whether results obtained with such a protocol can be transposed to everyday occurring EFE processing.

In recent years, there has been a growing interest for the analysis of ecological human behaviors during daily life interactions. It is especially the case for researchers concerned with realism, notably for pragmatic matters (Calvo and D’Mello, 2011). Unfortunately, the generalizability to ordinary emotional behaviors of mostly all results on EFE processing is unlikely because experimental methodologies lack ecological validity. Two key criticisms can be made. The first one concerns the stimuli. Research on the time course of EFE processing is undertaken with EFEs most often coming from the Pictures of Facial Affect database (POFA; Ekman and Friesen, 1976). The use of this dataset promotes comparison between studies and optimizes experimental conditions. However, these non-natural stimuli (EFEs of actors/actresses produced in non-natural contexts) are subjected to many criticisms (Tcherkassof et al., 2007). There are radical differences between non-natural behavioral stimuli (i.e., deliberate emotional displays) used in laboratory studies and those exhibited in everyday life (i.e., spontaneous emotional displays). Research on facial expression has highlighted how a crucial expressive feature of natural displays spontaneity is. Spontaneously occurring behavior differs in various aspects from deliberate behavior (Kanade et al., 2000), including timing and visual appearance (Hess and Kleck, 1990; Cohn and Schmidt, 2004). From an ecological perspective, affective analyses based on deliberate EFEs have a poor generalization capacity, which is why they need examples of naturally expressed EFEs and not prototypical patterns of facial behaviors such as POFA’s ones. Consequently, because they lack ecological validity, one questions the generalizability of experimental results that rely on unnatural stimuli. The second key criticism concerns the stimulus presentation. As mentioned above, participants are asked to fixate the center point of the stimulus which is displayed during a short time. However, this is far from an ordinary kind of activity. In an effort to overcome this issue, a free exploration paradigm using eye-tracking and EEG co-registration has been developed to evaluate the cognitive processing of stimuli in an ecological way. Eye-tracking methods are privileged means to examine the allocation of observers’ attention to different facial regions and to examine the relationship between gaze patterns and EFE processing (Schurgin et al., 2014). For joint EEG and eye movements recording, Kaunitz et al. (2014) extracted the eye fixation-related potentials (EFRPs) elicited by faces in a crowd. Simola et al. (2013, 2015) analyzed the eye movement-related brain responses to emotional scenes. Regarding EFEs processing more specifically, Neath-Tavares and Itier (2016) studied the co-registration of eye-tracking and EEG. However, they used a gaze-contingent procedure in order to test the diagnostic impact of different facial regions of interest (i.e., mouth, nose, left eye, right eye) in EFE processing. Hence, they did not study EFE processing in an ecological way since participants could not freely explore the stimuli but rather had specific regions of interest presented directly at their first and only fixation point. In any case, for joint EEG and eye movements recording, the use of both free eye-tracking and EEG co-registration in a free exploration paradigm raises questions as to estimating the evoked potential. When the stimulus is presented for a long duration and if the eye positions are not controlled to be stable, the usual estimation methodology by averaging is debatable: the potential estimated at the image onset not only reveals the potential directly elicited at the image presentation, but also the successive contributions of the visual information processing at each fixation rank. For example, in the case of the window’s latency of the LPP component (around 600–800 ms after the stimulus onset), it is reasonable to expect that one ocular fixation *before* this latency already occurred, even one fixation *during* this latency window also. In the study of Trautmann-Lengsfeld et al. (2013), the static stimulus was presented during 1,500 ms and the eye movements were not controlled. As a matter of fact, the neural activity observed during the latency window of the LPP component revealed not only the activity elicited by the stimulus presentation but also the activity provided by the early visual exploration of the stimulus with eye movements. This was in line with the objective of the Trautmann-Lengsfeld’s study which was the comparison between the perception of static and dynamic EFE. However, the stimuli in the Trautmann-Lengsfeld’s study weren’t spontaneous EFE but posed ones thus reducing the ecological significance of the results. Therefore, up to now, no study has yet examined the precise temporal dynamics of the EFE processing using eye-tracking and EEG co-registration with both ecological stimuli (i.e., freely expressed by ordinary people) and paradigm (i.e., free visual exploration). The present study aims at filling these data gaps.

The goal of our study is to study the EFE processing in ecological settings. We focused on unconstrained visual explorations (i.e., free eye movements) of natural emotional faces (spontaneous EFEs) contrary to what is usually done (i.e., fixed eye gaze and unnatural stimuli). As the Trautmann-Lengsfeld’s study, the stimuli in the present study were presented during a long time and the participants were free to visually explore them. This protocol is ecological from a visual exploration point of view both for dynamic and static stimuli, but used here for static stimuli. As a consequence, the methodology to estimate the evoked potentials has to be adapted to this experimental design. At the stimulus presentation and during the subsequent visual exploration by the ocular fixations, several cognitive processes are engaged. The estimation of the evoked potentials by averaging therefore fails to provide a reliable estimation of each of them because these brain responses overlap which each other. Consequently, in order to analyze the temporal dynamics of the EFE processing, a methodology is applied to distinguish between what is due to the stimulus presentation and what is due to its exploration. The precise objectives and hypotheses of the present study are as follow. Recent methodological studies on evoked potentials estimation have shown how promising are linear models decomposing the effects of different neural activities during a same temporal window (Burns et al., 2013; Bardy et al., 2014; Smith and Kutas, 2015a,b; Congedo et al., 2016; Kristensen et al., 2017a). This methodology is based on the General Linear Model (GLM). It is particularly suitable to estimate EFRPs and is more flexible (Kristensen et al., 2017b) than the ADJAR algorithm (Woldorff, 1993). This method has been recently implemented with success in EFRP/ESRPs (Eye Saccade-Related Potentials) estimation (Dandekar et al., 2012; Kristensen et al., 2017b). However, it has never been applied to the emotional field. Our aim is thus to exploit it for EEG activity, in order to examine the time course of the EFE processing during the very beginning of its visual exploration. Using the GLM, we hypothesize that it should be possible to deconvolve adjacent overlapping responses of the EFRPs elicited by the subsequent fixations and the ERPs elicited at the stimulus onset. If our hypothesis is correct, the time course of the early emotional processing could be analyzed through these estimated potentials. For this purpose, a joint EEG-eye tracking experiment was set up to take benefit on both synchronized experimental modalities (Dimigen et al., 2011; Nikolaev et al., 2016). Based on the valence hypothesis, the expected results are differences in ERPs’ amplitude depending on both valence and hemisphere (e.g., higher amplitude on left hemisphere for ERP components of positive emotions). According to the literature about the valence hypothesis (see for instance Graham and Cabeza, 2001; Wager et al., 2003; Alves et al., 2008), the effects should be located in the frontal regions. Moreover, and more importantly, based on articles by Noordewier and Breugelmans (2013), Noordewier et al. (2016), we expect that by analyzing the EFRPs of the first fixation, specific processes that might be valence-dependent will be differentiated.

## Materials and Methods

### Database

The DynEmo database (Tcherkassof et al., 2013) is a comprehensive resource of filmed affective facial behaviors which provides a substantial publicly available corpus of validated dynamic and natural facial expressions of pervasive affective states (i.e., representative of daily life affective expression). It supplies 358 EFE recordings performed by a wide range of ordinary people, from young to older adults of both genders (ages 25–65, 182 females and 176 males) filmed in natural but standardized conditions. DynEmo provides genuine facial expressions—or first-order displays—exhibited in the course of a given eliciting episode. The conditions that influence or cause affective behavior, whether internal or external to the expresser (her/his affective state, current situation, etc.), are known to the user. One-third of the EFE recordings have been displayed to observers who have rated (continuous annotations) the emotions displayed throughout the recordings. The dynamic aspects of these EFE recordings and their relationship to the observers’ interpretations are displayed in timelines. Such synchronized measures of expressing and decoding activities allow for a moment-by-moment analysis that simultaneously considers the expresser’s facial changes and the observer’s answers (independently of what is experienced by the expresser). Indeed, for each video, emotional expression timelines instantly signal when a given affective state is considered to be displayed on the face. Therefore, segmentation of the expressions into small emotion excerpts is easily achieved. For our study, the most expressive videos (maximum observers’ rates) were selected for four emotions (happiness, surprise, fear, and disgust). Then, inside each short EFE clip, the image corresponding to the maximum expression intensity (“apex”) was extracted. The final stimuli dataset was composed of 118 static EFE stimuli: neutral (24), happiness (12), surprise (12), fear (10), disgust (12), distractors (48), i.e., seventy target EFE stimuli with a controlled high recognition rate, and forty eight EFE diversion stimuli.

### Stimuli

**FIGURE 1:**
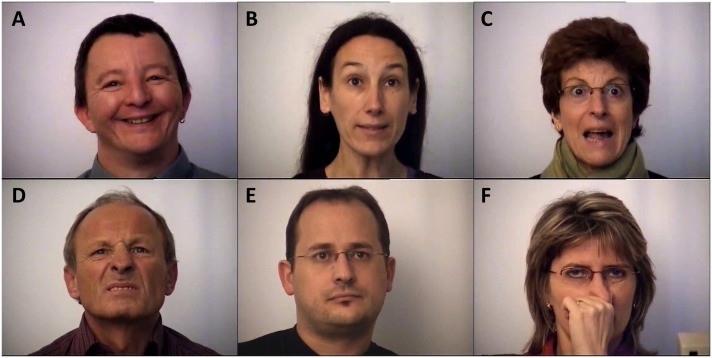
Example of EFE stimuli for each emotional category. **(A)** Happiness. **(B)** Surprise. **(C)** Fear. **(D)** Disgust. **(E)** Neutral. **(F)** Distractor.

Stimuli consist of 118 color EFE images, all equalized in luminance. The images’ resolution is 768 × 1024 pixels, subtended 30 × 40° of visual angle. **Figures 1A–F** illustrates one EFE stimulus for each emotional category. They were displayed onto a 20-inch ViewSonic CRT monitor located 57 cm in front of the participants of 768 × 1024 pixels and a 75 Hz refresh rate.

### Participants

Thirty-one healthy adults participated in the experiment, but data from only nineteen subjects (7 women and 12 men aged from 20 to 32 years – mean age 25 years 7 months (*SD* = 3 years 2 months, *SE* = 9 months) – were used for all the analyses. Data from six participants were discarded due to technical problems during acquisition (poor eye-tracking calibration, noisy EEG signals). Data from two participants were discarded due to high energy in the alpha band [8–12 Hz] in the occipital which is a criterion of a loss of attention. Finally, the data from two other participants were discarded because of a too low number of trials (see the Section “Brain Activity”). The remaining 19 participants were right-handed, except one male left-hander and one female left-hander. All participants had a normal or corrected-to-normal vision. They were free of any medical treatment at the time of the experiment, and had no history of neurological or psychiatric disorder. None of them had prior experience with the experimental task. All gave their written and informed consent prior to the experiment and were recompensed with 15€ in vouchers for their participation. The whole experiment was reviewed and approved by the ethics committee of Grenoble CHU (“Centre Hospitalier Universitaire”) (RCB: n° 2011-A00845-36). The co-registration EEG/Eye tracker was performed at the IRMaGe Neurophysiology facility (Grenoble, France).

### Experimental Protocol

Each run consisted of two separate and consecutive sessions. The eye movements and the EEG activity were recorded during both sessions. Note that only the recordings of the first session are presented here (the recordings of the second session being out of the scope of this article). In the first session, participants freely explored each static stimulus. To this end, they were asked to attentively watch the stimuli and to “empathize” with the displayed facial expressions. The 118 stimuli were randomly presented. The use of distractors in the first session aimed at preventing a memory effect on the emotional rating task carried out during the second session. Two short breaks were managed to avoid fatigue in participants. In a second session, participants had to rate each target EFE stimulus. The 70 target stimuli were presented in the same order than in the first session. Participants assessed the stimuli according to two scales: arousal on five levels, from -2 to 2 [not (--) to highly (++) arousing], and the emotional stimulus category (happiness, surprise, fear, disgust, and neutral). As this session was shorter, only one short break was introduced.

**FIGURE 2:**
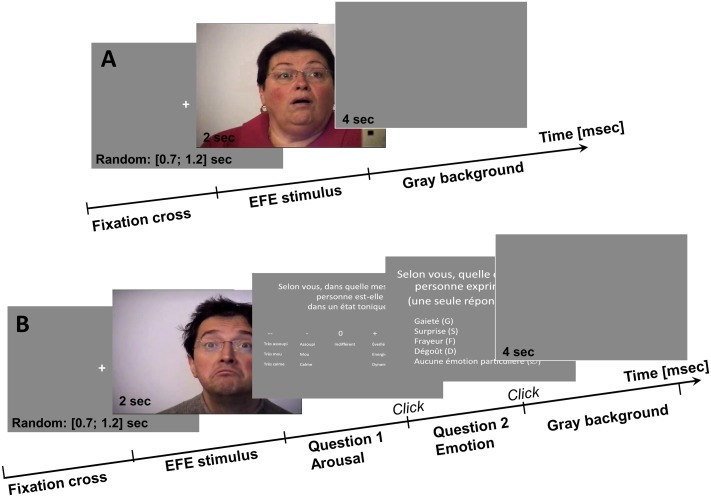
Temporal sequence of a trial, composed of different screens. The first session **(A)** was composed of 118 trials, and the second session **(B)** was composed of 70 trials with the two supplementary steps for the questions.

The timeline of the trials (**Figures 2A,B**) were similar between the two sessions (except for the two stimuli emotional ratings during the second session). Each trial started with the display of the fixation cross at the center of the screen, followed by the EFE stimulus displayed during 2 s, then the emotional ratings for the second session only (**Figure 2B**), and ended with a gray screen (4 s) before the next trial. The fixation cross was displayed on the center of the screen to initialize the exploration, during a random duration from 700 to 1,200 ms, to avoid the development of saccade anticipation before the visual stimulus presentation. The stimulus was displayed after the stabilization of the participant’s gaze on the fixation cross (during 500 ms before the end of its presentation, in a rectangle of 3° × 2° pixels around the fixation cross). During the second session, the display of the scales (arousal followed by emotion category) ended with the participant’s answer (key press). The trial terminated with a 4 s gray screen during which the participant could relax and blink.

For eye-tracking purpose, a 9-point calibration routine was carried out at the beginning of each session. It was repeated every 20 trials or when the drift correction, performed every 10 trials, reported an error above 1°. The complete experiment was designed thanks to the SoftEye software (Ionescu et al., 2009) to control (1) the timescale for the displays, (2) the eye-tracker and (3) the sending of synchronization triggers to both devices.

### Data Acquisition

#### Behavioral Measures

The behavioral data (EFEs’ arousal level and emotion categorization) were analyzed to determine how participants rated the pictures of the database. For the emotion categorization data, we used the index computation method of Wagner (1997). This author has recommended using an unbiased hit rate (Hu) when studying the accuracy of facial expression recognition to take into account possible stimulus and response biases. Wagner’s computation method combines the conditional probability that a stimulus will be recognized (given that it is presented) and the conditional probability that a response will be correct (given that it is used) into an estimate of the joint probability of both outcomes. This is done by multiplying together the two conditional probabilities divided by the appropriate marginal total (p. 50). Thus, the accuracy is a proportion of both responses and stimuli frequencies. Confusion matrixes are elaborated so that an unbiased hit rate (Hu) computed for each participant can be used as a dependent variable. The Hu ranges from 0 (no recognition at all) to 1 (complete recognition). Because the fear emotion was badly categorized (28% of the fear stimuli were recognized as surprise, and 16% of the fear stimuli were recognized as neutral), all data on the fear emotion were removed and analyses were conducted on four categories (Neutral, Disgust, Surprise, and Happiness). Moreover, the participants’ emotional categorizations during the second session were used as a ground truth to analyze data recorded during the first session. In other words, for a given participant, each EFE stimulus was re-categorized *post hoc* according to the emotion category the participant had assigned to the EFE. Thus, each participant had decoded a same emotion on slightly different subsets of stimuli. After decoding a given emotion, the associated subsets of stimuli (one subset per participant and per emotion) had large overlaps across participants, such as at least 50% of participants, in average, categorized 75% of same EFE stimuli into the same emotion. More precisely, this percentage of stimuli was distributed as follows across emotion: 75% for neutral, 75% for disgust, 50% for surprise and 100% for happiness.

The main argument supporting this re-categorization procedure is that encoding and decoding processes must not be confused, as stressed by Wagner (1997). Indeed, an encoder can express a given emotion when the decoder interprets this facial expression as displaying another emotion. As we are concerned by the decoding process, it justifies that we rely on the observer’s judgment rather than on the encoder’s emotion. This is especially relevant because the cerebral signals investigated are the ones corresponding to the observer’s own judgment. Let us recall that only EEG data from the first session are analyzed in this study. In this protocol, the neutral condition was the control condition, compared to the three other EFE (disgust, surprise, and happiness).

#### Ocular Activity

For the sake of compatibility with this EEG acquisition, the remote binocular infrared eye-tracker EyeLink 1000 (SR Research) was used to track the gaze of the guiding eye of each participant while he/she was looking at the screen. The EyeLink system was used in the Pupil-Corneal Reflection tracking mode sampling at 1,000 Hz. For eye-tracking acquisition purposes, the position of the head was stabilized with a chin rest.

**FIGURE 3:**
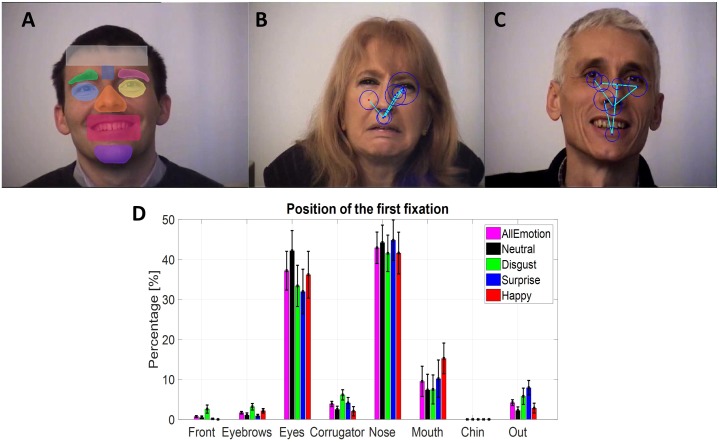
**(A)** Example of the masks to define the regions of interest in the EFE stimuli. **(B,C)** Two examples of scanpath for disgust and for happiness. **(D)** Mean percentage for the position of the first fixation depending on ROI and on emotion, based on individual means.

Eye gaze and EEG signals were synchronized offline on the basis of triggers sent simultaneously on both signals at each step of the trials, using the SoftEye software (Ionescu et al., 2009). Saccades and fixations were automatically detected by the EyeLink software. The thresholds for saccade detection were a minimum velocity at 30°/s, a minimum acceleration at 8000°/s^2^ and a minimum motion at 0.1°/s. In addition, specific triggers were added offline to each eye movement and EEG signals to indicate the beginning of the fixations depending on their localization in the EFE stimuli. Then, in order to select the fixations according to their spatial position, all EFE stimuli had been manually segmented into seven regions of interest (ROI), as illustrated in **Figure 3A**. The seven regions were the forehead, the left and right brows, the corrugator, the left and right eyes, the nose, the mouth, and the chin. An eighth region was added for fixations outside these regions.

#### Brain Activity

Participants’ electroencephalographic (EEG) activity was continuously recorded using an Acticap^®^ (Brain Products, Inc.) equipped with 64 Ag-AgCl unipolar active electrodes that were positioned according to the extended 10–20 system (Jasper, 1958; Oostenveld and Praamstra, 2001). The reference and ground electrodes used for acquisition were those of the Acticap, i.e., FCz for the reference, and AFz for the ground. The electro-oculographic (EOG) activity was also recorded using two electrodes positioned at the eyes outer canthi, and 2 respectively, above and below the left eye. Participants were free for their eye movements to explore the visual stimulus but they were instructed to limit blinking during the experimental session (see **Figures 3B,C**, for two examples of scanpath). Impedance was kept below 10 kΩ for all electrodes. The signal was amplified using a BrainAmp^TM^ system (Brain Products, Inc.) and sampled at 1,000 Hz with a 0.1 Hz high-pass filter and a 0.1 μV resolution. Data acquisition was performed using Grenoble EEG facility “IRMaGE.”

As regards EEG data preprocessing, the raw signal was first band-pass filtered between 1 and 70 Hz and a notch filter was added (50 Hz). The signals were visually inspected for bad channels. The rejected channels were interpolated. The signals were re-referenced offline to the average of all channels. Artifacts related to ocular movements (saccades and blinks) were corrected in a semi-automatic fashion using the signal recorded from the EOG electrodes and the SOBI algorithm (Belouchrani et al., 1997). The signal was then segmented into epochs that started 200 ms before and ended 2,000 ms after the image onset. Epochs were rejected when their variance exceeded a restrictive threshold of the mean variance across the epochs plus three standard deviations. Moreover, epochs were also rejected if there were less than two fixations during the 2-s trial. Data from participants without a minimum of five epochs per emotion were excluded. **Table 1** summarizes the number of epochs which were analyzed. EEGlab software (Delorme and Makeig, 2004) was used for all processing steps except the implementation of the GLM for the evoked potential estimation.

**Table 1:** Number of epochs per emotion based on individuals measures.

Number of trials	Neutral	Disgust	Surprise	Happiness
mean (*SE*)	19.3 (1.59)	12.4 (1.03)	12.9 (0.80)	12.2 (0.49)
[*min, max*]	[8, 32]	[5, 22]	[8, 20]	[8, 16]


Data were then baseline corrected according to the average EEG amplitude over the window from -200 ms to 0 ms before the image onset. Lastly, the signal from seven scalp regions (4/5 electrodes per region) was averaged to create seven virtual electrodes. These regions were evenly distributed across the scalp ranging from the frontal regions to the parieto-occipital ones, and from left to right, with the median occipital site also. These regions were defined as follows: left frontal (F3, F5, F7, FC5, FC3), right frontal (F4, F6, F8, FC6, FC4), left centro-parietal (C3, C5, T7, CP3, CP5), right centro-parietal (C4, C6, T8, CP4, CP6), left parieto-occipital (P3, P5, P7, PO3, PO7), right parieto-occipital (P4, P6, P8, PO4, PO8), and median occipital (POz, O1, Oz, O2).

### Estimation Methods

The two methods (Average and GLM) were applied on the same set of trials (**Table 1**), providing two estimations of the ERP at the stimulus onset, by averaging and by regression, and one estimation of EFRP by regression.

#### Estimation by Averaging

The estimation of evoked potentials by averaging time-locked EEG signals is the classical method. Let us note the signal *x_i_*(*t*) time-locked at the image onset during the *i^th^* epoch such as:

$$x_{i}(t)=s(t)+n_{i}(t)$$

with *s*(*t*) the potential evoked at the image onset and *n_i_*(*t*) the background cortical activity, considered as noise. Assuming that all stimuli elicit the same potential and that the ongoing activity is not synchronized to the fixation onset during the *i^th^* epoch, this potential is estimated by averaging on a given number of epochs as:

sAvg^(t) =1EΣi =1Exi(t).

It is well-known that this estimator is unbiased only if a unique potential is elicited per epoch (Ruchkin, 1965).

In our case, the EFE stimuli were categorized according to each participant’s own categorization. The estimation by average was done for each emotion for a given participant. Moreover during the latency of interest (from the image onset up to 600 ms), one or more fixations/saccades occurred (see the Section “Positions on the First Fixations”). Consequently the estimate sAvg^(t) is a biased estimation of the evoked potential at the image onset, but it is still an acceptable estimation for the global time-locked activity from the image onset. This global activity includes the activity elicited by the stimulus onset and the activity due to the visual exploration. The statistical results on sAvg^(t) are presented in the Section “Event Related Potential at the Image Onset Estimated by Averaging.” To separate these two neural activities, supplementary estimations were performed using the GLM, as explained further.

#### Estimation by Regression With the “General Linear Model”

The evoked potential at the image onset and the potentials elicited at each fixation and saccade rank overlapped one another. To take into account these response overlaps on the observed time-locked neural activity, a more accurate model can be designed such as:

$$x_{i}(t)=s(t)+{fp}^{\left(1\right)}(t-{\tau_{i}}^{\left(1\right)})+\overset{L\left(i\right)}{\underset{l=2}{\Sigma }}{fp}^{\left(2+\right)}\left(t-{\tau_{i}}^{\left(l\right)}\right)+\overset{L^{\prime }\left(i\right)}{\underset{l^{\prime }=1}{\Sigma }}sp\left(t-{{\tau_{i}}^{\prime }}^{\left(l^{\prime }\right)}\right)+n_{i}\left(t\right)$$

where *s*(*t*) is the evoked potential at the image onset, *fp*^(1)^(*t*) is the potential evoked at the first fixation rank, *fp*^(2+)^(*t*) the potential evoked at the second and following ranks, *sp*(*t*) the saccadic potential evoked at each saccade rank and *n_i_*(*t*) the noise of the ongoing activity. In this equation, for a given epoch *i*, ${\tau_{i}}^{\left(l\right)}$ is the timestamp of the fixation onset at rank *l*, and ${\tau_{i}}^{\prime \left(l^{\prime }\right)}$ is the timestamp at the saccade onset at rank *l’*. The justification of this model is the following:

- The potential elicited at the first fixation rank is *a priori* different from the one elicited at the following ranks. The rationale for this justification firstly comes from the oculomotor features which can be different at the very first fixation as compared to the followings when the exploration has already begun. Secondly, the categorization of the EFE depends on its recognition which is a fast process with a high contribution of the visual information processed at the first fixation rank (Batty and Taylor, 2003; Vuillemier and Pourtois, 2007).
- The saccadic activity is taken into account as this activity interacts with the early components of the EFRP at the posterior sites and also at the anterior sites (Nikolaev et al., 2016). Integrating these activities in the linear regression is a good solution (Dandekar et al., 2012). But, contrary to Dandekar et al.’s (2012) study, the saccadic potentials are not here the potentials of interest to analyze, but they are integrated into the model to provide unbiased estimations of the potentials of interest for this study which are mainly *s*(*t*) and *fp*^(1)^(*t*), and to a lesser extent *fp*^(2+)^(*t*).

By concatenating all trials, *s*(*t*) and *fp*^(1)^(*t*) are estimated by ordinary least square regression to obtain sGLM^(t) and fpGLM(1)^(t) namely. The statistical results on sGLM^(t) are presented in the Section “Event Related Potential at the Image Onset Estimated by Regression” and ones on fpGLM(1)^(t) and fpGLM(2+)^(t) in the Section “Eye Fixation Related Potentials Estimated by Regression.” Mathematical details for the GLM implementation concerning the selected configuration, as well as all configuration parameters for these estimations are given as Supplementary Material in Appendix 1.

### Statistical Analysis

For each participant, the averaged and regressed ERPs [sAvg^(t), sGLM^(t)] were separately computed per emotion condition and virtual electrode. On these evoked potentials, the mean amplitude of four components of interest, namely the P1, the N170, the P2–P3 complex that encompasses both the P2 and the P3 components (or the EPN which is the differential version with the neutral emotion) and the LPP were extracted. Using grand-average inspections, the windows used for the extraction of these amplitude data were adapted from that of Trautmann-Lengsfeld et al. (2013) to fit our data. The latency window for the P1 component was 90–130 ms post-stimulation. The latency window for the N170 component was 140–180 ms post-stimulation. The latency window for the P2–P3 complex was 200–350 ms post-stimulation. The latency window for the LPP component was 400–600 ms post-stimulation. Two components were extracted for the regressed EFRP: the Lambda response and the P2 component within a latency window of 20–100 ms, and 180–300 ms, respectively. In a first step, all these ANOVAs were performed using Statistica, had a 0.05 significance level, used Greenhouse–Geisser adjusted degrees of freedom when sphericity was violated (Significativity of the Mauchly’s test of sphericity) and were followed for each significant effect of a given factor by Tukey *post hoc* tests that corrected for multiple comparisons. Regarding the EFRPs, since there is no literature to which these results could be compared, we started by using *t*-tests against zero for the difference in component’s amplitude between each EFE and the neutral ones. In a second step, the statistical validity of the results was assessed to determine how the number of both participants and trials interact on each result (Boudewyn et al., 2017). This supplementary verification is undertaken because the number of participants and the number of trials per participant are in the lower range of the usual values. To do so, 1,000 experiments were simulated for each configuration given by a number of participants (N) and by a number of trials per participant and per emotion. The probability of observing the result is computed on average on all the simulated experiments (1,000) as a function of a given number of participants and a given number of trials per participant. Results are presented as Supplementary Material in Appendix 3.

## Results

### Behavioral Data

Arousal ratings for each EFE (**Table 2**) were statistically analyzed using a repeated measure ANOVA with emotion as within-participant factor. The main effect on emotion was significant [*F*(3,54) = 80.73, *p* < 0.0001, $\eta_{p}^{2}$ = 0.82]. The neutral EFEs (-0.29, *SE* = 0.13) elicited less arousal than disgust (0.73, *SE* = 0.11), surprise (0.70, *SE* = 0.07) and happiness (1.16, *SE* = 0.006) EFEs, which in return elicited more arousal than disgust (0.82, *SE* = 0.08) and surprise (0.75, *SE* = 0.07) EFEs.

**Table 2:** Mean arousal, unbiased hit rate, mean fixations number, and fixation duration (standard error in parentheses) depending on emotion, based on individual means.

	Neutral	Disgust	Surprise	Happiness
Arousal	-0.29 (0.13)	0.82 (0.08)	0.75 (0.07)	1.16 (0.06)
Hu rate	0.53 (0.04)	0.32 (0.03)	0.45 (0.04)	0.88 (0.03)
Fixations number	4.74 (0.21)	4.82 (0.23)	4.74 (0.21)	4.92 (0.20)
Mean fixation duration (ms)	301.91 (10.89)	297.54 (12.64)	293.12 (7.82)	287.31 (9.95)


The unbiased hit rate was computed (**Table 2**), based on the stimuli emotional categorization provided by the participant, and was statically analyzed using a repeated measure ANOVA with emotion as within-participant factor. The main effect on emotion was significant [*F*(3,54) = 67.38, *p* < 0.0001, $\eta_{p}^{2}$ = 0.79]. The unbiased hit rate was the lowest for disgust EFE (0.32, *SE* = 0.03), and was the highest for happiness EFE (0.88, *SE* = 0.03).

### Eye Movements’ Data

In this section, we first detail the global features of the eye movements’ data (the number of fixations and the average fixation duration for a complete trial) and then, more importantly, the specific features for the two first fixations. Results of the repartition of the fixation positions over the ROIs for the first fixation are presented. These results provide an external validation of the experimental data as they reproduce regular results on ocular positions associated to the EFE decoding. The results on the fixation duration, the fixation latency, the incoming saccade amplitude and orientation that are necessary elements for the configuration of the GLM, are detailed as Supplementary Material in Appendix 2.

#### Global Features

Both the number of fixations and the average fixation duration per trial (synthesized in **Table 2**) were statistically analyzed using two separated repeated measure ANOVAs with the emotion as within-participant factor. The fixations numbers were not different across emotion [*F*(3,54) = 1.24, *p* = 0.30, $\eta_{p}^{2}$ = 0.02], nor was the fixation duration [*F*(3,54) = 1.49, *p* = 0.23, $\eta_{p}^{2}$ = 0.08].

#### Positions on the First Fixations

The percentage in each spatial ROI regardless of the emotion (**Table 3**) was analyzed using a repeated measure ANOVA with the fixation rank and the ROI as within-participant factors. Only six ROIs were considered because the forehead and the chin ROIs were not enough fixated (respectively, 0.47% and 0.09%). As expected, a main effect on ROI was observed [*F*(5,90) = 37.5, *p* < 0.0001, $\eta_{p}^{2}$ = 0.68]: the eyes (41,94%, *SE* = 4.52%) and the nose (37.96%, *SE* = 3.76%) were the two ROIs the most fixated at the two first ranks. The rank by ROI interaction was significant [*F*(5,90) = 15.9, *p* < 0.0001, $\eta_{p}^{2}$ = 0.47] showing that the eyes were more fixated at the second fixation (47.7%, *SE* = 4.39%) than at the first fixation (37.19%, *SE* = 4.84%), and conversely the nose was most fixated at the first fixation (42.91,%, *SE* = 3.93%) than at the second fixation (33.02%, *SE* = 3.75%).

**Table 3:** Mean percentages (standard error in parentheses) in each ROI for the first and the second fixation, based on individual means.

Fixation	Eyebrows	Eyes	Corrugator	Nose	Mouth	Out
Rank 1	1.66 (0.33)%	37.2 (4.84)%	3.85 (0.66)%	42.9 (3.93)%	9.51 (3.78)%	4.17 (0.73)%
Rank 2	1.67 (0.47)%	46,7 (4.39)%	3.76 (0.94)%	33.0 (3.75)%	12.5 (2.83)%	1.89 (0.36)%


For the six most fixated ROIs, six separated ANOVAs (**Table 4**) were run to analyze specifically the position of the first fixation (**Figure 3D**) according to the emotion (within-participant factor).

**Table 4:** Mean percentages (standard error in parentheses) in each ROI, depending on emotion, for the first and the second fixation, based on individual means.

	Neutral	Disgust	Surprise	Happiness	ANOVA
Eye brows	1.06 (0.57)%	3.15 (0.81)%	0.89 (0.37)%	2.14 (0.57)%	***F*(3,54) = 3.22, *p* = 0.029, $\eta_{p}^{2}$ = 0.15; ^∗^**
		**Disgust > Surprise (^∗^)**			
Eyes	42.2 (5.04)%	33.4 (5.15)%	32.0 (5.58)%	36.2 (5.84)%	*F*(3,54) = 2.59, *p* = 0.061, $\eta_{p}^{2}$ = 0.13
Corrugator	2.50 (0.79)%	6.12 (1.29)%	4.12 (1.36)%	2.03 (1.16)%	*F*(3,54) = 2.60, *p* = 0.061, $\eta_{p}^{2}$ = 0.13
		Disgust > Happiness (trend)			
Nose	44.2 (4.36)%	41.5 (4.56)%	44.8 (5.07)%	41.6 (5.22)%	*F*(3,54) = 0.30, *p* = 0.82, $\eta_{p}^{2}$ = 0.016
Mouth	7.35 (3.93)%	7.49 (3.63)%	10.2(4.68)%	15.2 (3.84)%	***F*(3,54) = 4.52, *p* = 0.006,$\eta_{p}^{2}$ = 0.20; ^∗∗^**
		**Happiness > Neutral ∼ Disgust (^∗^)**			
Out	2.22 (0.90)%	5.77 (2.03)%	7.91 (1.81)%	2.82 (1.22)%	***F*(3,54) = 2.84, *p* = 0.046, $\eta_{p}^{2}$ = 0.14; ^∗^**
		Surprise > Neutral (trend)			


*^∗^*p* < 0.05, ^∗∗^*p* < 0.01, bold: significant effect.*

For the mouth ROI, a significant larger percentage on this ROI was observed for the happiness emotion (15.24%, *SE* = 3.84%) compared to the disgust (7.49%, *SE* = 3.63%) and neutral (7.35%, *SE* = 3.93%) emotions. A significant difference was observed for the eyebrows ROI, with a larger percentage of first fixation on this ROI for the disgust emotion (3.15%, *SE* = 0.81%) than for the surprise emotion (0.89%, *SE* = 0.37%). For the corrugator ROI, a trend was observed with a larger percentage on this ROI for the disgust emotion (6.12%, *SE* = 1.29%) compared to the happiness emotion (2.03%, *SE* = 1.16%). Finally, a trend was observed for the percentage of the first fixation outside the ROIs, larger for the surprise emotion (7.91%, *SE* = 1.81%) than for the neutral one (2.22%, *SE* = 0.90%).

### Brain Activity

#### Event-Related Potential at the Image Onset Estimated by Averaging

**FIGURE 4:**
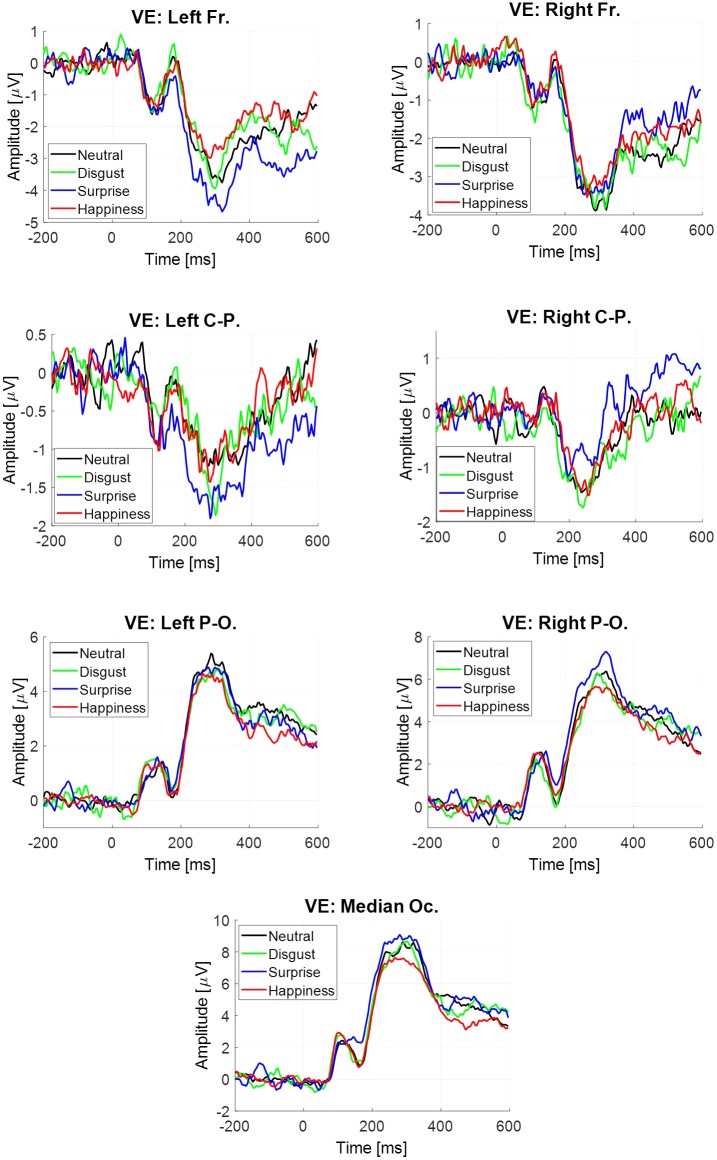
Event-related potentials elicited at the stimulus onset estimated by averaging, depending on emotion and virtual electrode.

The estimate sAvg^(t) was obtained for each participant, each emotion and each virtual electrode (**Figure 4**). Four components were extracted. All statistical results are noticed in **Table 5**. Only significant effects are detailed below.

**Table 5:** Statistical results of the ANOVAs performed on the evoked potential at the image onset, estimated by averaging.

Estimation Avg	Evoked potential at the image onset		
	
	Virtual electrode (VE)	Emotion (EMO)	VE × EMO
P1 [90–130] ms	***F*(6,108) = 14.41; *p* < 0.001; $\eta_{p}^{2}$ = 0.44; ^∗∗∗^**	*F*(3,54) = 1.22; *p* = 0.31; $\eta_{p}^{2}$ = 0.06	*F*(18,324) = 0.88; *p* = 0.64; $\eta_{p}^{2}$ = 0.03
	**Fr. > CP. > PO, Oc.**		
N170 [140–180] ms	***F*(6,108) = 3.02; *p* = 0.009; $\eta_{p}^{2}$ = 0.14; ^∗∗^**	*F*(3,54) = 2.36; *p* = 0.08; $\eta_{p}^{2}$ = 0.11	***F*(18,324) = 1.78; *p* = 0.026; $\eta_{p}^{2}$ = 0.09; ^∗^**
	**Fr. > C.P. > P.O., Oc.**		
P2–P3 [200–350] ms	***F*(6,108) = 54.86; *p* < 0.001; $\eta_{p}^{2}$ = 0.75; ^∗∗∗^**	*F*(3,54) = 2.08; *p* = 0.11; $\eta_{p}^{2}$ = 0.10	***F*(18,324) = 2.95; *p* < 0.001; $\eta_{p}^{2}$ = 0.14; ^∗∗∗^**
	**Fr. > C.P. > P.O., Oc.**		**Left Fr. : H > S**
LPP [400–600] ms	***F*(6,108) = 38.85; *p* < 0.001; $\eta_{p}^{2}$ = 0.68; ^∗∗∗^**	*F*(3,54) = 0.77; *p* = 0.51; $\eta_{p}^{2}$ = 0.04	***F*(18,324) = 2,15; *p* = 0.005; $\eta_{p}^{2}$ = 0.11; ^∗∗^**
	**Fr. > C.P. > P.O., Oc.**		**Left Fr. : H > S**


*^∗^*p* < 0.05, ^∗∗^*p* < 0.01, ^∗∗∗^*p* < 0.001, bold: significant effect.*

There was a significant main effect of the virtual electrode for the P1 component, the N170 component, the P2–P3 complex and the LPP (**Table 5**). After *post hoc* decomposition and Tukey corrections, no differences were significant for the N170 component. For each of the three other components, significant differences were observed with higher mean amplitudes at posterior sites than at central sites which were higher in return than at anterior sites.

For both the P2–P3 complex and the LPP, the interaction between emotion and virtual electrode was significant (**Table 5**). For the P2–P3 complex, the happiness condition lead to a higher mean amplitude (-2.27 μV, *SE* = 0.59 μV) than for the surprise condition (-3.67 μV, *SE* = 0.59 μV) at the left frontal site. For the LPP, its mean amplitude was higher in the happiness condition (-1.56 μV, *SE* = 0.39 μV) than in the surprise condition (-3.05 μV, *SE* = 0.62 μV) at the left frontal site. Monte Carlo simulations (see Supplementary Material in Appendix 3) were performed on the LPP extracted from the ERP at the stimulus onset estimated by averaging. It confirmed that this difference on the LPP was present at the left frontal site and absent at the right frontal site.

#### Event-Related Potential at the Image Onset Estimated by Regression

**FIGURE 5:**
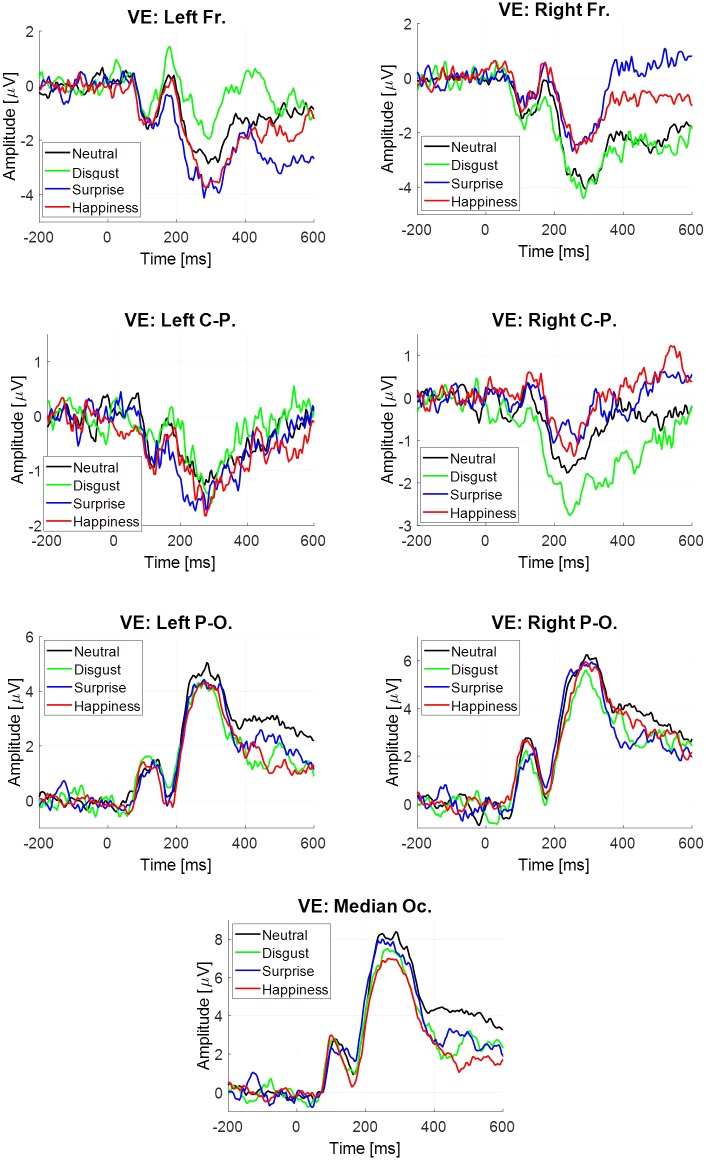
Event-related potentials elicited at the stimulus onset estimated by regression, depending on emotion and virtual electrode.

The estimate sGLM^(t) was obtained for each participant, each emotion and each virtual electrode (**Figure 5**). Four components were extracted. All statistical results are noticed in **Table 6**.

**Table 6:** Statistical results of the ANOVAs performed on the evoked potential at the image onset, estimated by regression.

Estimation GLM	Evoked potential at the image onset		
	
	Virtual electrode (VE)	Emotion (EMO)	VE × EMO
P1 [ 90–130] ms	***F*(6,108) = 13.28; *p* < 0.001; $\eta_{p}^{2}$ = 0.42; ^∗∗∗^**	*F*(3,54) = 1.17; *p* = 0.33; $\eta_{p}^{2}$ = 0.06	*F*(18,324) = 0.85; *p* = 0.63; $\eta_{p}^{2}$ = 0.04
	**Fr. > C.P. > P.O., Oc.**		
N170 [140–180] ms	*F*(6,108) = 1.98; *p* = 0.07; $\eta_{p}^{2}$ = 0.10	*F*(3,54) = 0.64; *p* = 0.59; $\eta_{p}^{2}$ = 0.03	*F*(18,324) = 1.07; *p* = 0.39; $\eta_{p}^{2}$ = 0.06
P2–P3 [200–350] ms	***F*(6,108) = 27.30; *p* < 0.001; $\eta_{p}^{2}$ = 0.60; ^∗∗∗^**	*F*(3,54) = 1.19; *p* = 0.32; $\eta_{p}^{2}$ = 0.06	*F*(18,324) = 0.99; *p* = 0.47; $\eta_{p}^{2}$ = 0.05
	**Fr. > C.P. > P.O., Oc.**		
LPP [400–600] ms	***F*(6,108) = 8.83; *p* < 0.001; $\eta_{p}^{2}$ = 0.33; ^∗∗∗^**	*F*(3,54) = 1.06; *p* = 0.37; $\eta_{p}^{2}$ = 0.06	*F*(18,324) = 1.09; *p* = 0.057; $\eta_{p}^{2}$ = 0.06
	**Fr. > C.P. > P.O., Oc.**		Right Fr. : D < S (trend)


*^∗∗∗^*p* < 0.001; bold, significant effect; Fr. for left and right frontal sites; C.P. for left and right centro-parietal sites; P.O., Oc. for left and right parieto-occipital sites and median occipital site; Left Fr. for left frontal site; H for Happiness, S for Surprise.*

For all components except N170, there was a significant main effect of virtual electrode (**Table 6**), with a higher mean amplitude of both components at posterior sites than at anterior sites. For the LPP, only a trend difference was observed in the disgust condition with a lower amplitude (-2.39 μV, *SE* = 0.79 μV) than in the surprise condition (0.54 μV, *SE* = 1.69 μV) at the right frontal site.

No significant modulation across emotion was observed based on the neural activity estimated on sGLM^(t), while a modulation was observed based on the neural activity estimated on sAvg^(t). Then, the objective of Monte Carlo simulations realized on the LPP extracted from sGLM^(t) (see Supplementary Material in Appendix 3) was to assess the absence of such a modulation (happiness vs. surprise). It confirmed that if this difference between these two EFEs was present on the LPP at the left frontal site on the neural activity sAvg^(t), this difference was definitively absent on the LPP at the left and right frontal sites on the neural activity sGLM^(t).

#### Eye Fixation-Related Potentials Estimated by Regression

**FIGURE 6:**
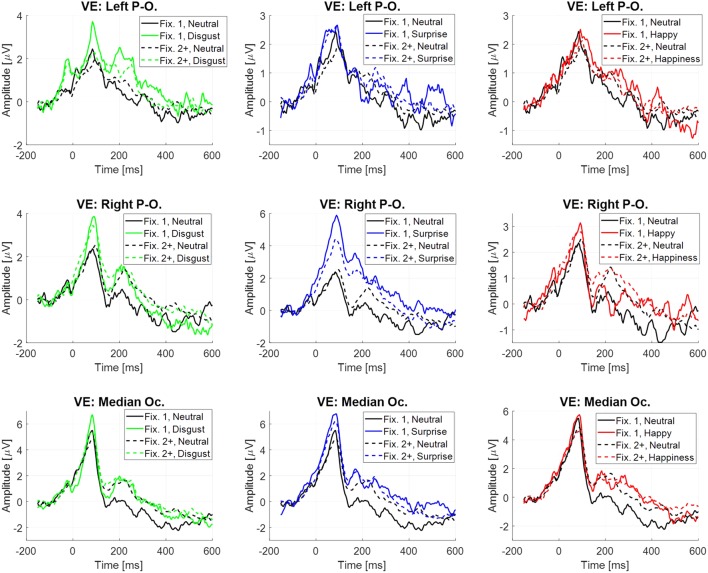
Eye fixation-related potentials elicited at the first fixation onset (plain line) and at the following ranks (dotted line) estimated by regression on the right parieto-occipital site (top), left parieto-occipital site (middle) and median occipital site (bottom), depending on emotion, from left to right: disgust vs. neutral, surprise vs. neutral and happiness vs. neutral.

The difference between the two previous estimations for the evoked potential at the stimulus onset is the inclusion or not of the neural activity linked to fixations. This activity through EFRP estimation is analyzed in this section, and is focused on emotional stimuli compared to neutral, at the occipital sites (VE: Left Parieto-occipital, Right Parieto-occipital and Median Occipital). Two EFRPs were estimated by the GLM: the first at the first fixation onset, namely fpGLM(1)^(t) and the next one at the second and following fixation onsets, namely fpGLM(2+)^(t) (**Figure 6**). Two components were extracted, the lambda response between 20 and 100 ms, and the P2 component between 180 and 400 ms. The mean amplitude of each component was analyzed using a repeated measure ANOVA with the fixation rank and the emotion as within-participant factors. All statistical results are given in **Table 7**. Only significant effects are detailed below.

**Table 7:** Statistical results of the Student’s tests performed on the EFRPs, estimated by regression.

Estimation GLM	Evoked potential at the first fixation onset		
	
	*Disgust – Neutral*	*Surprise – Neutral*	*Happiness – Neutral*
***Left parieto-occipital site***		
Lambda [20–110] ms	*t*(18) = 0.49; *p* = 0.63; $\eta_{p}^{2}$ = 0.01	*t*(18) = 1.25; *p* = 0.23; $\eta_{p}^{2}$ = 0.08	*t*(18) = 0.02; *p* = 0.98; $\eta_{p}^{2}$ = 0
P2 [180–400] ms	*t*(18) = 1.28; *p* = 0.22; $\eta_{p}^{2}$ = 0.09	*t*(18) = 0.58; p = 0.57; $\eta_{p}^{2}$ = 0.02	*t*(18) = 0,66; *p* = 0.52; $\eta_{p}^{2}$ = 0.02
***Right parieto-occipital site***		
Lambda [20–110] ms	*t*(18) = 0.60; *p* = 0.55; $\eta_{p}^{2}$ = 0.02	***t*(18) = 2.67; *p* = 0.016; $\eta_{p}^{2}$ = 0.30; ^∗^**	*t*(18) = 0.32; *p* = 0.76; $\eta_{p}^{2}$ = 0.006
P2 [180–400] ms	*t*(18) = 0.73; *p* = 0.48; $\eta_{p}^{2}$ = 0.03	***t*(18) = 3.07; *p* = 0.007; $\eta_{p}^{2}$ = 0.36; ^∗∗^**	*t*(18) = 0.62; *p* = 0.54; $\eta_{p}^{2}$ = 0.02
***Median occipital site***		
Lambda [20–110] ms	*t*(18) = 0.17; *p* = 0.87; $\eta_{p}^{2}$ = 0.002	*t*(18) = 1.53; *p* = 0.14; $\eta_{p}^{2}$ = 0.12	*t*(18) = 0.26; *p* = 0.79; $\eta_{p}^{2}$ = 0.004
P2 [180–400] ms	*t*(18) = 1.08; *p* = 0.29; $\eta_{p}^{2}$ = 0.06	***t*(18) = 2.25; *p* = 0.037; $\eta_{p}^{2}$ = 0.23; ^∗^**	*t*(18) = 1.32; *p* = 0.20; $\eta_{p}^{2}$ = 0.09
**Estimation GLM**	**Evoked potential at the second and following fixation onsets**		
***Left parieto-occipital site***		
Lambda [20–110] ms	*t*(18) = 0.76; *p* = 0.46; $\eta_{p}^{2}$ = 0.03	*t*(18) = 1.22; *p* = 0.24; $\eta_{p}^{2}$ = 0.08	*t*(18) = 0.26; *p* = 0.80; $\eta_{p}^{2}$ = 0.004
P2 [180–400] ms	*t*(18) = 0.74; *p* = 0.47; $\eta_{p}^{2}$ = 0.03	*t*(18) = 0.57; *p* = 0.58; $\eta_{p}^{2}$ = 0.02	*t*(18) = -0.01; *p* = 0.99; $\eta_{p}^{2}$ = 0
***Right parieto-occipital site***		
Lambda [20–110] ms	*t*(18) = 0.91; *p* = 0.38; $\eta_{p}^{2}$ = 0.05	*t*(18) = 1.72; *p* = 0.10; $\eta_{p}^{2}$ = 0.15	*t*(18) = 0.48; *p* = 0.64; $\eta_{p}^{2}$ = 0.01
P2 [180–400] ms	*t*(18) = 0.18; *p* = 0.86; $\eta_{p}^{2}$ = 0.002	*t*(18) = 1.24; *p* = 0.23; $\eta_{p}^{2}$ = 0.08	*t*(18) = 0.71; *p* = 0.49; $\eta_{p}^{2}$ = 0.03
***Median occipital site***		
Lambda [20–110] ms	*t*(18) = 0.36; *p* = 0.72; $\eta_{p}^{2}$ = 0.01	*t*(18) = 1.03; *p* = 0.32; $\eta_{p}^{2}$ = 0.06	*t*(18) = 0.03; *p* = 0.98; $\eta_{p}^{2}$ = 0
P2 [180–400] ms	*t*(18) = 0.48; *p* = 0.64; $\eta_{p}^{2}$ = 0.01	*t*(18) = 0.79; *p* = 0.44; $\eta_{p}^{2}$ = 0.03	*t*(18) = 0.87; *p* = 0.40; $\eta_{p}^{2}$ = 0.04


*^∗^*p* < 0.05, ^∗∗^*p* < 0.01; bold, significant effect; Fr. for left and right frontal sites; C.P. for left and right centro-parietal sites; P.O., Oc. for left and right parieto-occipital sites and median occipital site.*

Significant differences were observed on the first EFRP and between surprise and neutral. A significant difference was observed on the right parieto-occipital site for the Lambda response, with a higher amplitude for surprise (4.39 μV, *SE* = 1.20 μV) than for neutral (1.82 μV, *SE* = 0.70 μV). For the P2 component, there was a significant difference at the right parieto-occipital site with a higher mean amplitude for surprise (1.40 μV, *SE* = 0.44 μV) than for neutral (-0.39 μV, *SE* = 0.40 μV). A significant difference was also found for the P2 component between surprise (0.93 μV, *SE* = 0.63 μV) and neutral (-0.89 μV, *SE* = 0.64 μV) at the median occipital site. It is known that the local physical features of a visual stimulus influence the amplitude of the Lambda response (Gaarder et al., 1964). The statistical results showed that there was no difference across emotion for the local standard deviation of the luminance on the region gazed at the first fixation, nor any difference across emotion of the local luminance through the first saccade. These results are presented as Supplementary Material in Appendix 2. Moreover, the Monte Carlo simulation (see Supplementary Material in Appendix 3) confirmed that differences were present at the right parieto-occipital site and at the median occipital site for the first EFRP, and were absent for the second and subsequent EFRP.

## Discussion

The goal of the present study was to analyze the temporal dynamics of spontaneous and static emotional faces decoding. More precisely, the early visual exploration’s temporal dynamics of natural EFEs was explored. Eye movements and EEG activities were jointly recorded and analyzed. Recent studies using such joint recordings have shown the interest of a regression approach based on the GLM to estimate ERPs as well as eye fixation/saccade related potentials (Dandekar et al., 2012; Kristensen et al., 2017b). The overlapping evoked potentials can be separately estimated by deconvolution when using this method. Consequently, these methodological tools take the temporal dimension into account. This is particularly interesting for the study of the dynamics of EFE processing. For instance, Noordewier et al. (2016) have stressed the importance of taking the temporal dimension into account to understand the nature of surprise. Thus, the contribution of this study is twofold. First, it addresses naturally occurring human affective behavior. Second, it offers a solution to the methodological issue regarding the estimation of overlapping evoked potentials.

Based on an ecological approach, this study used natural EFEs as static stimuli and a free exploration task. Natural EFEs are spontaneous expressions encountered in everyday life and free exploration is an ecological paradigm requiring the consideration of time for analysis. Behavioral results on eye movements are consistent with what is usually observed when studying emotional facial features processing. Since the 1920s, there is evidence that specific facial features, such as the eyes and mouth, are relevant for the decoding of EFEs (Buchan et al., 2007; Schurgin et al., 2014). Present results showed that the eyes and the nose were the two most gazed ROIs at the first fixation, irrespective of the emotion displayed. The former ROI is in accordance with usual results as the eyes are very important for social interaction to decode the emotional state of the other person. As regard to the nose, results are interpreted as an exposition bias. The fixation cross allowing the gaze stabilization before the EFE presentation was at the center of the image, thus close to the nose position in the face. The third most gazed ROI was the mouth which is also an important region for emotion decoding. This region was gazed more for the happiness emotion than for the disgust and neutral emotions at the first fixation. This is also in line with previous research. When looking at happy facial expressions, participants usually fixate the mouth region for a longer time (e.g., Eisenbarth and Alpers, 2011). Eyebrows are likely to be diagnostic features as well. Observers gazed significantly more at this area when looking at EFEs of disgust as compared to EFEs of surprise. They also tended to gaze more at the corrugator area for EFEs of disgust than EFEs of happiness for instance. On the whole, areas of the face attracting attention more than other areas were quite in line with what is usually observed (Beaudry et al., 2014; Vaidya et al., 2014). Finally, when facing EFEs of surprise, observers tended to collect information out of the face as if they were trying to find in the environment what could have caused such an emotion.

The other key contribution of this study concerns the methodological issue to estimate overlapping evoked potentials. This is a main concern in synchronized EEG and eye movement analysis (Dimigen et al., 2011), and more specifically here as the time was an important issue for the free exploration task. It has been well-established that the estimation of evoked potentials by averaging time-locked EEG signals is biased in the case of overlapping responses. Woldorff (1993) proposed an iterative procedure in the context of ERP experiments where the EEG signal is time-locked on external events. It was called the ADJAR algorithm, and was designed to estimate overlap responses from immediately adjacent events, to converge toward the evoked potential of interest. Moreover, regression techniques, especially the GLM (Kiebel and Holmes, 2003), have proved their efficiency in the estimation of evoked overlapping potentials (Dale, 1999; Dandekar et al., 2012; Burns et al., 2013; Bardy et al., 2014; Kristensen et al., 2017a). Besides, the ADJAR algorithm appears to be poorly suited to EFRP estimation (Kristensen et al., 2017a). In this respect, a regression-based estimation of evoked potentials was done (Smith and Kutas, 2015a,b). Usually, the linear model is designed either with the saccade onset timestamps as regressors (Dandekar et al., 2012), either with the fixation onset timestamps as regressors (Kristensen et al., 2017b). In our study, both types of regressors were integrated into the same model. A third type was added, namely the timestamp of the stimuli onset. The rationale for such a model, with both the timestamps of saccade and fixation onsets, was the observation of different distributions for incoming saccade amplitudes and orientations depending on emotions. It is well known that the saccadic activities just before the fixation onsets modulate the early component (specially the Lambda response) of each EFRP. Thus, to provide an unbiased estimator of the EFRPs from these confounding factors, the timestamps of saccade onsets were added to the model. Moreover, the timestamps of the fixation onsets were split into two different classes, those for the first fixation, and those for the following fixations. This way, the EFRP for the first fixation was discriminated from the EFRP for the second and subsequent fixations. The timestamps of the stimulus onset were finally also added because the main objective of this study was to distinguish, from the whole neural activity, the one specifically elicited by the stimulus during a given latency window. Altogether, this study shows how the GLM can be adapted to a specific issue and how its configuration plays a central role in the methodological approach.

We also focused on the neural activity during the latency of the P2–P3 complex and of the LPP. It was analyzed with regards to the estimations comparison: Average vs. GLM. The common estimation by averaging takes into account all neural activities time-locked at the stimulus onset. It is commonly accepted that the potential evoked at a visual stimulus presentation lasts about 700 ms, corresponding to the time needed for the stimulus-evoked activity to fade (Dimigen et al., 2011; Nikolaev et al., 2016). This potential had the largest contribution in the neural activity during the latency of the P2–P3 complex ([200; 350] ms). Moreover, its contribution was larger for the P2–P3 latency than for the LPP latency ([400; 600] ms). This evoked potential was also estimated by the GLM. The neural activity provided by the free exploration of the stimulus explained the difference between these two estimates. Indeed, the average fixation latency was about 250 ms for the first one and about 500 ms for the second one. We will first discuss these differences on the potential elicited at the stimulus onset (ERPs), before focusing on the evoked potentials at the first fixations (EFRPs).

Regarding the cerebral responses to the natural EFEs, two different activation patterns were observed for the ERPs computed by the averaging on the one hand and by the GLM on the other hand. For the former (Average), the estimated evoked potential includes the potential at the stimulus onset and the activation provided by the visual exploration of the ocular fixations. For the later (Regression), the estimated evoked potential takes only into account the potential elicited at the stimulus onset. As expected, the amplitude of both the P2–P3 complex and the LPP was higher at posterior sites than at anterior sites for both methods, in accordance with the classical topographical distribution of these components. However, for the averaging method there was a low activation pattern (negative) at the left frontal site with a higher amplitude for both components for the happiness than the surprise condition. Rather for the GLM method, no significant modulation across emotions was observed (only a trend at the right frontal site with the amplitude of the LPP, lower for the disgust than the surprise condition which will be discussed below). The discrepancy between the two methods’ results is easily explained. Indeed, for the averaging method, the activation amplitude included both the potential at the stimulus onset and the activation provided by the visual exploration on the ocular fixations. And for the GLM method, the estimated activation included only the potential at the stimulus onset. Therefore, the frontal left negative pattern observed using the averaging method might in fact only rely upon the activations linked to the subsequent fixations and not on the activation from the stimulus onset.

Concerning the hemispheric prevalence, for the time window of the P2–P3 complex and of the LPP, a higher amplitude of the neural activity was observed for the happiness emotion compared to the surprise emotion when the neural activity was estimated by averaging on time-locked signals at the stimulus presentation. The prevalence of the left electrode site is in accordance with the valence hypothesis, with the involvement of the subsequent fixations for discriminating the happiness emotion as compared to others. The left hemisphere would be preferentially dedicated to the analysis of positive emotions such as happiness (Reuter-Lorenz and Davidson, 1981; Adolphs et al., 2001). When using the GLM to analyze the “common” ERPs (i.e., without the involvement of the subsequent fixations), the only trend differences (*p* = 0.057) between EFEs were found at the right frontal site: the cerebral response to the disgust emotion was enhanced compared to the other ones. This is also in line with the valence hypothesis which posits a right hemispheric specialization for negative affects, such as disgust (Reuter-Lorenz et al., 1983). Yet with common ERP, no difference was found on the left frontal site, but only a trend difference on the right frontal site. That means that the perception of EFEs at the stimulus onset might possibly be firstly mostly undertaken by the right hemisphere (Indersmitten and Gur, 2003; Davidson et al., 2004; Tamietto et al., 2006; Torro Alves et al., 2008). And then, the impact of the subsequent fixations that reveal the involvement of the left hemisphere might therefore reveal the bilateral gain advocated by Tamietto et al. (2006), and in a more general manner, the predominance of the right hemisphere at the stimulus onset and, afterward, the implication of the left hemisphere for the subsequent fixations. It would illustrate the “complex and distributed emotion processing system” detailed by Killgore and Yurgelun-Todd (2007). Hence, it seems that the recruitment of the left hemisphere needs to be primed by a first analysis performed by the right hemisphere. The communication that would take place to ensure such a bilateral recruitment as soon as the first fixation occurs, as well as any causal link, still need to be further explored using spectral and connectivity analyses. Yet, as reported by Tamietto et al. (2007), neuroimaging studies have already shown that the structures involved in EFE processing are various homologous regions of both hemispheres, such as the early sensory cortices, the middle prefrontal cortex and subcortical areas like the amygdala. They also detail that interhemispheric communication might occur at the early stages through connections at the level of the limbic system, while later processing steps allow for an interhemispheric communication through the corpus callosum.

As to the time course of EFE processing, when computing the ERPs by averaging and as expected, we found emotion-dependent modulations of the amplitude of the P2–P3 complex as well as the LPP component. Yet, no difference was found between EFEs for the N170, which might be in favor of the part of the literature that views the first stage as a raw structural processing one (Eimer et al., 2003), which might also be linked to stimuli of low arousal (Almeida et al., 2016). Considering the ERPs computed using the regression method, no significant impact of emotion on the brain response elicited exclusively by the presentation of the stimuli was found. This difference with the literature might be explained by the stimuli and paradigm we used. Indeed, Neath-Tavares and Itier (2016), like most authors interested in this research topic, use prototypical stimuli (whether from POFA or from the MacBrain Face Stimulus Set, Batty and Taylor, 2003). This might explain at least in part why we do not have the same impact of valence on EFEs decoding as revealed by ERPs. In fact, with prototypical stimuli, the displayed emotions are overstated and amplified, whereas in the present study, EFE are natural and spontaneous, thus weaken (Tcherkassof et al., 2013; cf. also Wagner et al., 1986; Valstar et al., 2006). For prototypical stimuli, the actors exaggerate the EFE. For instance, some past studies showed that posed smiles are larger in amplitude and are longer in duration than spontaneous smiles (Ekman and Friesen, 1982; Cohn and Schmidt, 2004; Schmidt et al., 2006). Valstar et al. (2006) also showed that characteristics of brow actions (as such as intensity, speed and trajectory) are different between spontaneous and posed EFE. In our case, the filmed persons expressed spontaneously and naturally the EFE. Consequently, we used less “intensified” or “aroused” EFE than other studies based on prototypical EFE (Tcherkassof et al., 2013). Another explanation is that, when the participants freely explore a stimulus, the brain responses to the presentation of the stimulus can be polluted by the subsequent responses to saccades that can occur after only 200 ms post-stimulation. In our case, in addition to using natural stimuli, we analyzed separately the brain responses elicited by the stimulus presentation only and the subsequent fixations. Hence, since with natural stimuli and our unconstrained paradigm we found no significant modulation of components’ amplitude when using the ERPs computed using the regression method, it might be that the arousal of the used EFEs was too low.

Finally, with respect to EFRPs, the early potential called Lambda response was impacted by the EFE presentation: the amplitude of the Lambda response was significantly higher for the surprise EFE than for the neutral EFE over the right parieto-occipital site. The Lambda response reflects the visual change in the image retina due to the saccade (Yagi, 1979). This response is modulated by low-level visual features as luminance and contrast across the saccade but also by the by the amplitude and orientation of the saccade (Gaarder et al., 1964; Hopfinger and Ries, 2005; Ossandón et al., 2010). High level factors such as task demand and information processing load also modulate the lambda amplitude (Yagi, 1981; Ries et al., 2016). Since low-level factors have entirely been taken into account in this experiment (i.e., global luminance equalization for the stimuli, local luminance verification at the first fixation and saccadic response estimated by the GLM), high-level factors might indeed explain this impact on the Lambda response.

Furthermore, a difference on the P2 component evaluated on the first EFRP was observed between surprise and neutral emotions over the right parieto-occipital and the median occipital site. The visual P2 component is known to be involved in many different cognitive tasks (Key et al., 2005), such as visual feature detection (Luck and Hillyard, 1994). It is modulated by numerous factors like attention allocation, target repetition, task difficulty, but also by the emotional content of faces (Stekelenburg and de Gelder, 2004) and an interaction between valence and arousal was found on EFRP at the visual exploration of emotional scenes (Simola et al., 2015). In our study, the fact that this effect was observed only for the surprise emotion is interesting. The valence of the surprise may be positive or negative depending on the context (Noordewier and Breugelmans, 2013). This effect may be linked to the areas the participant gazed at the first fixation when displayed surprise EFE: a higher number of fixations tended to land out of the selected face ROIs (forehead, eye brows, corrugator, eyes, nose, mouth, and chin) for surprise EFE as compared to neutral EFE. It is as if participants needed to extract information out of the faces to decode the displayed EFE in order to find cues what could have caused such an emotion. This interpretation has to be studied deeper with dedicated experiments. However, this modulation of the first EFRP with the surprise emotion compared to the neutral emotion contributes to the activation pattern of the LPP on the evoked potential at the stimulus onset estimating by averaging, as mentioned above. Lastly, the fact that such a difference only occurs between the surprise and the neutral conditions for the EFRPs cannot rule out completely an impact of arousal for this particular physiological marker. In line with Simola et al. (2015), such an interaction between valence and arousal for fixation-related potentials would be particularly interesting to study in the EFE processing context.

The present investigation is a promising initial work for the study of emotional decoding’s time course. More participants and more trials need to be run to strengthen this exploratory work. Yet, it appears that the visual exploration of emotional faces is a critical ingredient of EFE processing. It is especially the case when stimuli are not prototypical displays, as in ordinary life. For an accurate comprehension of the displayed emotion, observers need to look through the face, and even outside the face. That is why research on facial behavior urgently requires a dynamic approach (Fernández-Dols, 2013). Moreover, the dynamic propriety of EFE is a key feature of facial behavior since it consists of facial features dynamically shifting. The method presented here is an auspicious tool to treat the decoding of this dynamic information. Such work is currently undertaken by the authors.

## Author Contributions

AT was responsible for the emotion part of this research. AG-D was responsible for the engineering part of this research. LV was responsible for the experimental procedure. RR, AG-D, and EK collected the data. AG-D, RR, EK, and BR are in charge of the data analysis.

## Conflict of Interest Statement

The authors declare that the research was conducted in the absence of any commercial or financial relationships that could be construed as a potential conflict of interest.
