# Supplementary material for: Temporal Dynamics of Natural Static Emotional Facial Expressions Decoding: A Study Using Event- and Eye Fixation-Related Potentials
Source: Front Psychol. 2018 Jul 12;9:1190. doi: 10.3389/fpsyg.2018.01190 (PMC6052106; doi:10.3389/fpsyg.2018.01190)
Supplement: Supplementary file 1 [file Data_Sheet_1.pdf]

Supplementary Material

Temporal Dynamics of Natural Emotional Facial Expressions Decoding: A study using Event- and Eye Fixation-Related Potentials

Anne Guérin-Dugué[1†], Raphaëlle N. Roy[2], Emmanuelle Kristensen[1,5], Bertrand Rivet[1], Laurent Vercueil[3,4], Anna Tcherkassof[5]

[1] Univ. Grenoble Alpes, CNRS, Grenoble INP*, GIPSA-lab, 38000 Grenoble, France

* Institute of Engineering Univ. Grenoble Alpes

[2]ISAE-SUPAERO, Univ. Fédérale de Toulouse, France

[3]Univ. Grenoble Alpes, Inserm, CHU Grenoble Alpes, GIN, 38000 Grenoble, France

[4]EFSN, PPNR, CHU Grenoble Alpes, 38043 Grenoble France

[5] Univ. Grenoble Alpes, Univ. Savoie Mont Blanc, LIP/PC2S, Grenoble, France

[†] **Corresponding Author**
anne.guerin@gipsa-lab.grenoble-inp.fr

1 Appendix 1: GLM configuration

This supplementary material describes the configuration and the implementation of the GLM to estimate the evoked potentials of interest: the evoked potential at the stimulus presentation, at the first fixation rank and at the subsequent ranks. The saccadic potentials elicited at the saccade onset (whatever the rank) was also considered, as the distribution of the incoming saccades, in terms of direction and amplitude, differed across emotions. Let us recall the equation:

$$x_i(t) = s(t) + fp^{(1)}\left(t - \tau_i^{(1)}\right) + \sum_{l=2}^{L(i)} fp^{(2+)}\left(t - \tau_i^{(l)}\right) + \sum_{l'=1}^{L'(i)} sp\left(t - \tau_i'^{(l')}\right) + n_i(t)$$

where $s(t)$ is the evoked potential at the image onset, $fp^{(1)}(t)$ is the potential evoked at the first fixation rank, $fp^{(2+)}(t)$ the potential evoked at the second and following ranks, $sp(t)$ the saccadic potential evoked at each saccade rank and $n_i(t)$ the noise of the ongoing activity. In this equation, for a given epoch i, $\tau_i^{(l)}$ is the timestamp of the fixation onset at rank l, and $\tau_i'^{(l')}$ is the timestamp at the

saccade onset at rank l'. The potentials $s(t)$, $fp^{(1)}(t)$, $fp^{(2+)}(t)$ and $sp(t)$ are estimated by ordinary least square regression. This equation can be rewritten in matrix form:

$$\forall i \in \{1, \dots, E\}, \qquad \boldsymbol{x_i} = \boldsymbol{D}_s . \boldsymbol{s} + \boldsymbol{D}_{fp,i}^{(1)} . \boldsymbol{fp^{(1)}} + \boldsymbol{D}_{fp,i}^{(2+)} . \boldsymbol{fp^{(2+)}} + \boldsymbol{D}_{sp,i} . \boldsymbol{sp_c} + \boldsymbol{n}_i .$$

$\boldsymbol{x}_i$ is the vector ($\boldsymbol{x}_i = [x_i(1), \dots, x_i(N_e)]^\dagger$; $\boldsymbol{x}_i \in \mathbb{R}^{N_e}$) of the observed EEG samples time-locked to stimulus onset, for the ith epoch, with $[.]^\dagger$ the transpose operator. N_e is the number of samples, that is, the length of the observed signal $x_i(t)$. $\boldsymbol{n}_i$ is the noise vector ($\boldsymbol{n}_i = [n_i(1), \dots, n_i(N_e)]^\dagger$; $\boldsymbol{n}_i \in \mathbb{R}^{N_e}$) with the same number of samples. $\boldsymbol{s} \in \mathbb{R}^{N_s}$ is the vector of the evoked potential at the stimulus onset, and N_s is the length of this potential. $\boldsymbol{fp^{(1)}} \in \mathbb{R}^{N_{fp}}$ is the vector of the potential evoked at the first fixation onset and N_{fp} is the length of this potential. $\boldsymbol{fp^{(2+)}} \in \mathbb{R}^{N_{fp}}$ is the vector of the potential evoked at the second and subsequent fixation onset and N_{fp} is the length of this potential. $\boldsymbol{sp} \in \mathbb{R}^{N_{sp}}$ is the vector of the saccadic response time-locked to saccade onset irrespective to the rank of the saccade onset in the epoch and N_{sp} is the length of this potential. $\boldsymbol{D}_s \in \mathbb{R}^{N_e \times N_s}$ is the Toeplitz matrix[1] with N_e rows and N_s columns, for stimulus onset. $\boldsymbol{D}_s$ is defined by its first column, with entries that are all equal to zero except for one at the row subscript corresponding to the temporal position 0 ms. $\boldsymbol{D}_{fp,i}^{(1)} \in \mathbb{R}^{N_e \times N_{fp}}$ is the Toeplitz matrix with N_e rows and N_{fp} columns, for coding the first fixation onset during the ith epoch. $\boldsymbol{D}_{fp,i}^{(1)}$ is defined by its first column, with entries that are all equal to zero except one, at the row subscript corresponding to the temporal $\tau_i^{(1)}$ ms, in the ith epoch. $\boldsymbol{D}_{fp,i}^{(2+)} \in \mathbb{R}^{N_e \times N_{fp}}$ is the Toeplitz matrix with N_e rows and N_{fp} columns, for coding the second and subsequent fixation onsets during the ith epoch. $\boldsymbol{D}_{fp,i}^{(2+)}$ is defined by its first column, with entries that are all equal to zero except the entries at the row subscript corresponding to the temporal positions $\tau_i^{(l)}, l > 1$ ms, in the ith epoch. $\boldsymbol{D}_{sp,i} \in \mathbb{R}^{N_e \times N_{sp}}$ is the Toeplitz matrix with N_e rows and N_{sp} columns, for coding all saccade onsets during the ith epoch. $\boldsymbol{D}_{sp,i}$ is defined by its first column, with entries that are all equal to zero except the entries at the row subscript corresponding to the temporal positions $\tau_i'^{(l')}, l' > 0$ ms, in the ith epoch. Unlike the three other matrices, the $\boldsymbol{D}_s$ matrix does not depend on the epoch number (i), as the timestamps of stimulus onset are always equal to zero whatever the epoch. The four Toeplitz matrices are sparse matrices. Matrices $\boldsymbol{D}_s$ and $\boldsymbol{D}_{fp,i}^{(1)}$ are composed of only one diagonal equal to one, all other values being equal to zero. For the matrices $\boldsymbol{D}_{fp,i}^{(2+)}$ and $\boldsymbol{D}_{sp,i}$ the number of diagonals (equal to one) corresponds respectively to the number of fixations minus one, or to the number of saccades, in the ith epoch. All other values are equal to zero. Considering all epochs (E), the observations are concatenated such that:

$$\boldsymbol{x} = \boldsymbol{D}_S . \boldsymbol{s} + \boldsymbol{D}_{Fp}^{(1)} . \boldsymbol{fp^{(1)}} + \boldsymbol{D}_{Fp}^{(2+)} . \boldsymbol{fp^{(2+)}} + \boldsymbol{D}_{Sp} . \boldsymbol{sp} + \boldsymbol{n}$$

[1] By definition, a Toeplitz matrix is a descending diagonal-constant matrix.

with $\underline{x} = [\underline{x}_1^\dagger, \dots, \underline{x}_{N_e}^\dagger]^\dagger \in \mathbb{R}^N$ $\boldsymbol{x} = \left[\boldsymbol{x}_1^\dagger, \dots, \boldsymbol{x}_E^\dagger\right]^\dagger \in \mathbb{R}^N$, $\boldsymbol{D}_S = \left[\boldsymbol{D}_S^\dagger, \dots, \boldsymbol{D}_S^\dagger\right]^\dagger \in \mathbb{R}^{N \times N_s}$, $\boldsymbol{D}_{Fp}^{(1)} = \left[\boldsymbol{D}_{fp,1}^{(1)\dagger}, \dots, \boldsymbol{D}_{fp,E}^{(1)\dagger}\right]^\dagger \in \mathbb{R}^{N \times N_{fp}}$, $\boldsymbol{D}_{Fp}^{(2+)} = \left[\boldsymbol{D}_{fp,1}^{(2+)\dagger}, \dots, \boldsymbol{D}_{fp,E}^{(2+)\dagger}\right]^\dagger \in \mathbb{R}^{N \times N_{fp}}$ and $\boldsymbol{D}_{Sp} = \left[\boldsymbol{D}_{sp,1}^\dagger, \dots, \boldsymbol{D}_{sp,E}^\dagger\right]^\dagger \in \mathbb{R}^{N \times N_{sp}}$, where $N = N_e \times E$,$N = N_e \times E$ N is the total number of samples. After concatenation of the Toeplitz matrices and evoked potentials, this equation becomes $\boldsymbol{x} = \boldsymbol{D}.\boldsymbol{p} + \boldsymbol{n}$ with $\boldsymbol{D}$ equal to $[\boldsymbol{D}_S, \boldsymbol{D}_{Fp}^{(1)}, \boldsymbol{D}_{Fp}^{(2+)}, \boldsymbol{D}_{Sp}]$ and $\boldsymbol{p}$ is the concatenation of the four evoked potentials such that $\boldsymbol{p} = \left[\boldsymbol{s}^\dagger, \boldsymbol{fp}^{(1)\dagger}, \boldsymbol{fp}^{(2+)\dagger}, \boldsymbol{sp}^\dagger\right]^\dagger$. The solution given by the least square minimization is:

$$\widehat{\boldsymbol{p}_{GLM}} = (\boldsymbol{D}^\dagger.\boldsymbol{D})^{-1}.\boldsymbol{D}.\boldsymbol{x}$$

where $\widehat{\boldsymbol{p}_{GLM}}$ is the concatenation of all estimates, such that $\widehat{\boldsymbol{p}_{GLM}} = \left[\widehat{\boldsymbol{s}_{GLM}}^\dagger, \widehat{\boldsymbol{fp}_{GLM}^{(1)}}^\dagger, \widehat{\boldsymbol{fp}_{GLM}^{(2+)}}^\dagger, \widehat{\boldsymbol{sp}_{GLM}}^\dagger\right]^\dagger$.

As for the estimation by averaging, the model was applied separately for each participant and each emotion. The grand average was then obtained by averaging all estimates for all participants and each emotion.

The main configuration parameters for the GLM are (1) the time intervals for the estimated potentials and (2) the time interval of the epoch for the observed signal $x(t)$, providing the number of samples[2] (i.e. N_s, N_{fp} and N_{sp}) for the evoked potentials at the stimulus onset, at the fixation onset, at the saccade onset and the number of samples for the observed signal (i.e. N_e). The estimation window of the potential $s(t)$ extended from 200 ms (baseline computation between -200 and 0 ms) before stimulus onset to 900 ms after. It was a duration long enough to estimate this transient response at the stimulus presentation. Thus, the total duration for the potential $s(t)$ was 1100 ms, thereby defining the number N_s of samples. The estimation window of the potentials $fp^{(1)}(t)$ and $fp^{(2+)}(t)$ extended from 150 ms (baseline computation between -150 and -50 ms) before the fixation onset to 900 ms after. The total duration for the EFRPs was 1050 ms, thereby defining the number N_{sp} of samples. The estimation window for the saccadic potential $sp(t)$ was configured from 50 ms (baseline computation between -50 and -10 ms) before saccade onset to 300 ms after. The total duration for the ESRPs was 350 ms, thereby defining the number N_{sp} of samples. The epoch duration for the observed signal $x(t)$ was set from 200 ms before text onset to 1830 ms after. This value was chosen as the sum of the length of the EFRP (900 ms) with the latency of the third fixation (+ 790 ms) and two times its standard deviation (2 ×70 ms) to ensure. The number N_e of samples of the observed signals was then defined.

[2] The number of samples is the duration of the time interval multiplied by the sampling frequency.

2 Appendix 2: Ocular and visual features on the first fixations

Supplementary Table 1 synthesizes the ocular features for the first fixations. The durations of the first and the second fixation were statistically analyzed using a repeated measure ANOVA with the fixation rank (1 vs 2) and the emotion as within-participant factors. A main effect of rank was observed ($F(1,18) = 36.75$, $p < 0.0001$, $\eta p^2 = 0.67$), and the second fixation duration (299.50 ms, se = 12.69 ms) was longer than the first one (229.41 ms, se = 8.87 ms). The differences according to emotions were not significant ($F3,54) = 2.06$, $p = 0.116$, $\eta p^2 = 0.10$).

The latency of the first fixation was between 248 ms and 267 ms in average depending on the emotion. The neural activity between 600 and 800 ms (latency window of the LPP component) was impacted by the visual information perception at the first fixation rank. The latency of the second fixation was in average between 488 ms and 536 ms depending on the emotion, just before the latency window of the LPP component.

Supplementary Table 1: Mean features (standard error in parentheses) for the first and the second fixation/saccade, depending on the emotion, based on individual means: the fixation duration, the fixation latency and the incoming saccade amplitude

	Neutral	*Disgust*	*Surprise*	*Happiness*
		First rank		
Fixation duration [ms]	242.73 (9.37)	227.84 (12.51)	212.63 (8.54)	234.42 (11.86)
Fixation latency [ms]	267.00 (9.18)	255.54 (9.92)	248.00 (9.50)	263.08 (11.56)
Incoming saccade amplitude [°]	1.95 (0.08)	2.18 (0.12)	2.03 (0.10)	1.94 (0.10)
		Second rank		
Fixation duration [ms]	309.04 (18.00)	306.94 (18.07)	300.79 (14.40)	281.26 (10.87)
Fixation latency [ms]	536.71 (12.45)	509.47 (13.83)	488.29 (11.46)	525.63 (14.48)
Incoming saccade amplitude [°]	1.85 (0.11)	2.06 (0.11)	2.08 (0.10)	2.11 (0.12)

The amplitudes of the first and the second incoming saccade were statistically analyzed using a repeated measure ANOVA with the fixation rank (1 vs 2) and the emotion as within-participant factors. Only a main effect of emotion was observed ($F(3,54) = 4.82$, $p = 0.005$, $\eta p^2 = 0.21$). For the first two incoming saccades, the amplitude was in average larger for the disgust EFE (2.12°, se = 0.09°) than for the neutral EFE (1.90°, se = 0.09°).

Concerning the orientation of the incoming saccade (Supplementary Figure 1), Kolmogorov-Smirnov tests were performed for each pair of different emotions (Supplementary Table 2). For each emotion, orientation data were collected for all trials and all subjects at the first saccade rank. For each pair of emotions, the statistical distributions of the incoming saccade orientation were different, except for the pair disgust vs happiness. The same procedure was repeated for the second and subsequent ranks. For each pair of emotions, the statistical distributions of the incoming saccade orientation were different, except for the disgust vs happiness, and neutral vs surprise pairs. Finally, the orientation distributions for each saccade rank regardless of the emotion were not different. Regardless the emotion, the two

distributions for the incoming saccade amplitude at the first rank and at the second and subsequent ranks were not significantly different. These results confirmed the choice of the GLM, including the estimation of the saccadic potential. This is especially true for the orientation distributions.

Supplementary Table 2: Kolmogorov-Smirnov test to compare the orientation distributions of the first incoming saccade and the second one, depending on emotion, *p<0.05, **p<0.01, bold: significant effect

	Disgust	***Surprise***	***Happiness***
		First rank	
Neutral	**d_{ks} = 0.275, p < 0.001, ****	**d_{ks} = 0.162, p = 0.0006, ****	**d_{ks} = 0.274, p < 0.0001, ****
Disgust		**d_{ks} = 0.202, p = 0.0001, ****	d_{ks} = 0.114, p = 0.086
Surprise			**d_{ks} = 0.252, p < 0.0001, ****
		Second rank	
Neutral	**d_{ks} = 0.182, p = 0.0001, ****	d_{ks} = 0.060, p = 0.624	**d_{ks} = 0.241, p < 0.0001**
Disgust		**d_{ks} = 0.162, p = 0.002, ****	d_{ks} = 0.072, p = 0.548
Surprise			**d_{ks} = 0.199, p = 0.0001, ****
		All emotions	
		First rank	
Second rank		d_{ks} = 0.047, p = 0.162	

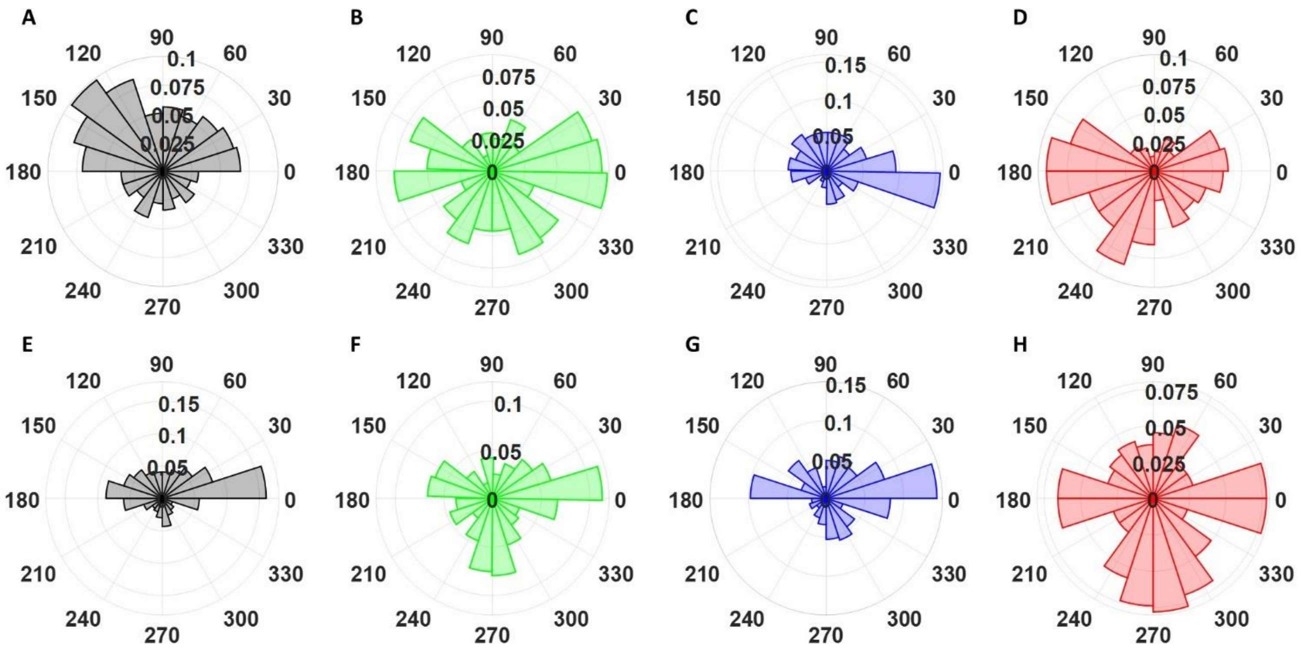

Supplementary Figure 1: Polar histogram of the orientation of the incoming saccade for each emotion (A, E: Neutral; B, F: Disgust; C, G: Surprise and D, H: Happiness, for the first saccade (Top) and the second and subsequent ones (Bottom)

It is known that the local physical features of a visual stimulus influence the amplitude of the Lambda response (Gaarder et al., 1964). According to Ossandon and collegues, there is a strong correlation between the peak amplitude of the lambda sub-component (latency around 100 ms) and the absolute difference of the local luminance at the start position and the end position of a saccade. For the first saccade and the first fixation, this difference is computed by the difference of the local luminance at the first fixation minus the luminance of the gray screen presented just before the stimulus. The Supplementary Table 3 summarizes the statistics on local luminance difference across the first saccade positions and the local RMS contrast at the first fixation position.

Supplementary Table 3: Mean luminance features (standard error in parentheses) for the first saccade and fixation, depending on the emotion, based on individual means: the absolute value of the luminance difference across the first saccade and the local RMS contrast at the first fixation position.

Luminance	***Neutral***	***Disgust***	***Surprise***	***Happiness***
Local difference	39.43 (1.49)	37.02 (2.00)	34.42 (1.92)	42.85 (1.22)
Local RMS contrast	23.75 (0.40)	23.40 (0.42)	23.66 (0.58)	23.60 (0.51)

The absolute difference of the local luminance across the first saccade positions and the local RMS contrast evaluated by the standard deviation of the local luminance at the first fixation position were statistically analyzed by two separated repeated measure ANOVAs with emotion as within-participant factor. On the local luminance difference, the main effect on emotion was significant ($F(3,54) = 7.48$, $p = 0.0003$, $\eta p^2 = 0.29$). On average, the local luminance difference across the first saccade positions was higher for neutral stimuli as compared to the surprise stimuli.

For the local RMS contrast at the first fixation position, no significant difference was observed across emotions ($F(3,54) = 0.12$, $p = 0.95$, $\eta p^2 = 0.95$).

3 Appendix 3: Monte Carlo simulations

To assess the statistical validity of our results, we have used the proposed methodology by Boudewyn and colleagues.

This methodology was applied on the evoked potentials estimated by averaging and by GLM. It is expected that the estimation by GLM needs more data to be reliable than the estimation by averaging, because more information is estimated with a same amount of observations for the former.

To assess the statistical validity of the results, 1000 experiments were simulated for each configuration given by a number of participants (N) and by a number of trials per participant and per emotion. The number of trials for the simulated experiments increased from 4 to 22 trials. When this number was greater than the effective number of trials for a given emotion and a given participant, all trials were selected.

Let us first consider the estimation of the Event Related Potential at the image onset estimated by averaging. In the real experiment, a significant difference at the left frontal site was observed for the LPP between surprise and happiness conditions. This result was assessed with the Monte Carlo simulations. The evolution of the probability for achieving this difference as a function of the number of trials and the number of participants, is illustrated at the Supplementary Figure 2. The absence of any effect between happiness and surprise at the right frontal site was also assessed.

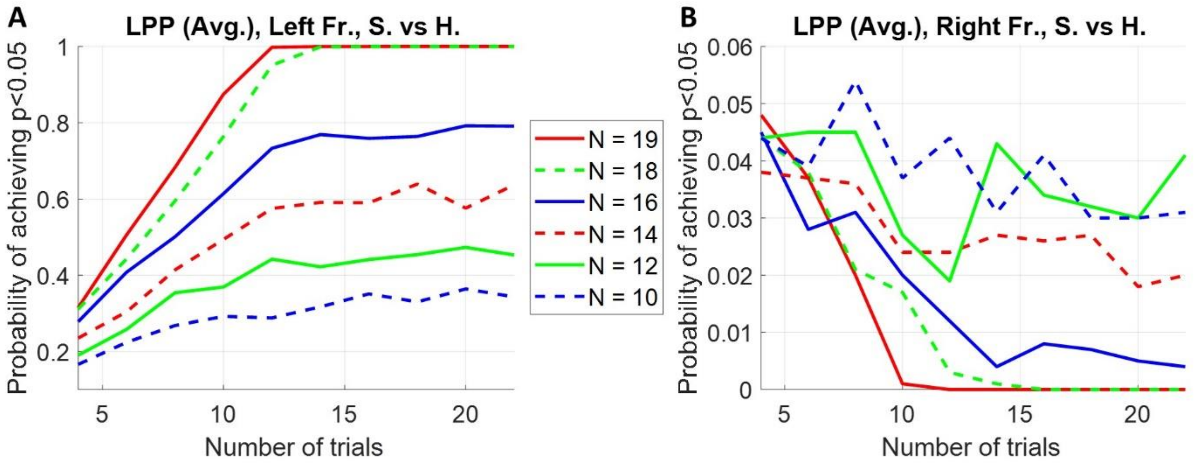

Supplementary Figure 2: Probability for achieving the significant difference on the LPP of the ERP at the EFE onset, estimated by averaging, between the surprise and happiness conditions, at the left frontal site (A), and at the right frontal site (B)

Similarly, the absence of any effect between happiness and surprise on the LPP from the evoked potential at the stimulus onset estimated by the GLM was assessed with the same methodology. The results are illustrated below on the left and right frontal sites (Supplementary Figure 3)

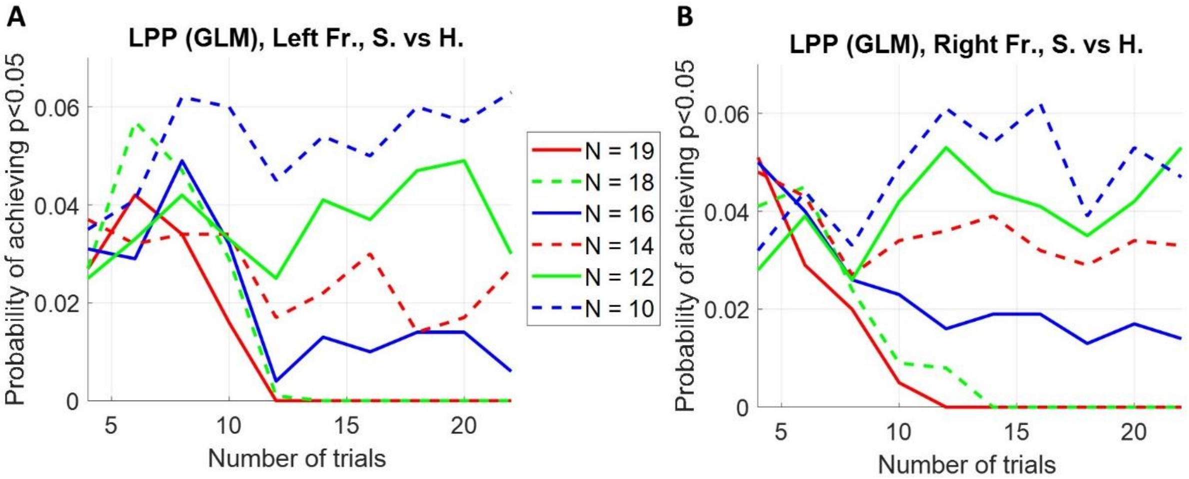

Supplementary Figure 3: Probability for achieving the significant difference on the LPP of the ERP at the EFE onset, estimated by GLM, between the surprise and happiness conditions, at the left frontal site (A) and at the right frontal site (B)

For the first EFRP, a significant difference was founded between surprise and neutral 1) on the right parieto-occipital site for the Lambda response and for the P2 component, and 2) on the median occipital site for the P2 component only. We tested these three effects as well as the absence of difference between surprise and neutral on the median occipital site for the Lambda response (Supplementary Figure 4). As a comparison, these four effects were also assessed with this methodology for the second EFRP (Supplementary Figure 5).

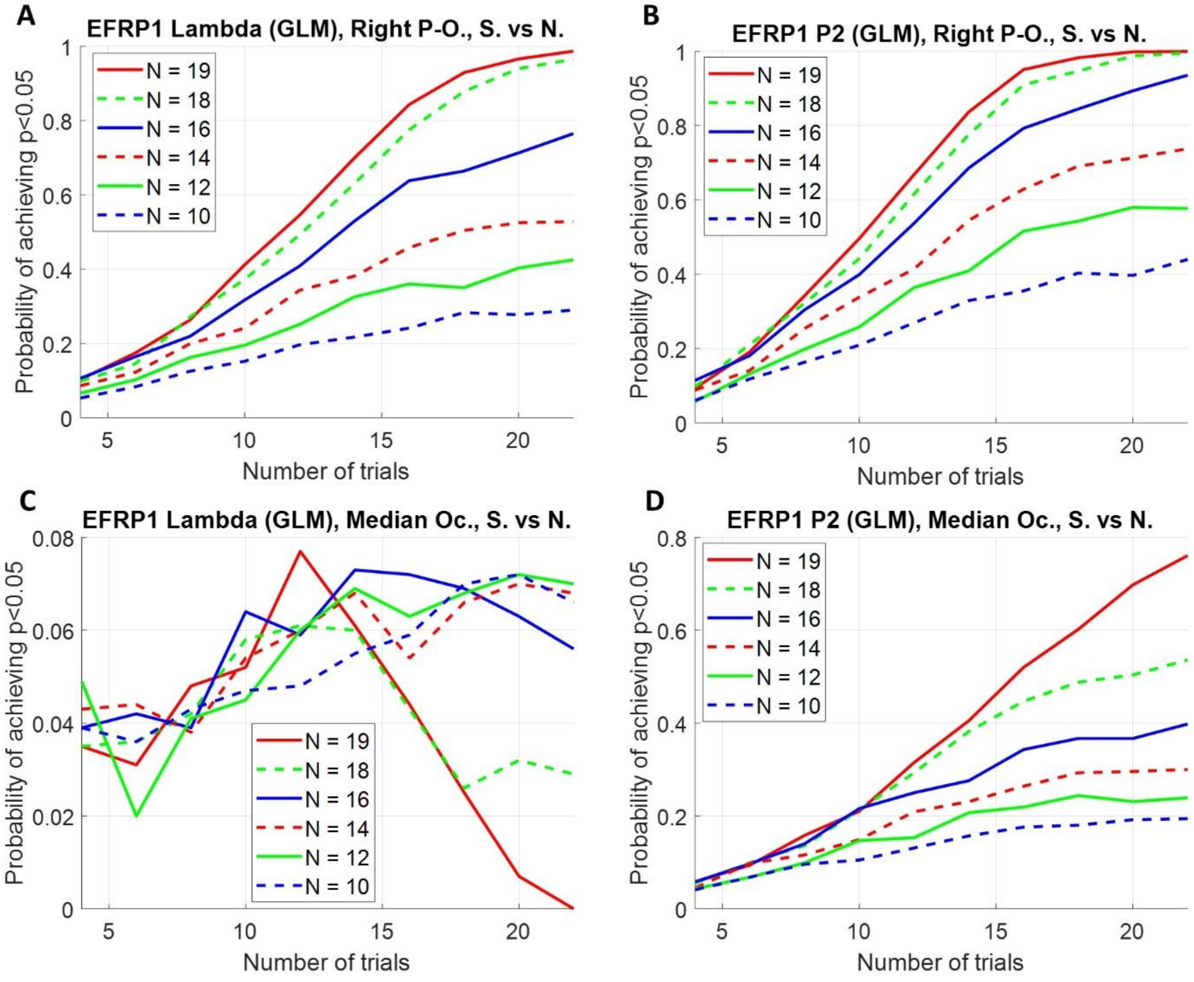

Supplementary Figure 4: Probability for achieving the significant difference on Lambda response (A, C) and the P2 component (B, D) of the first EFRP, between surprise and neutral conditions, at the right parieto-occipital site (A, B) and at the median occipital site (C, D)

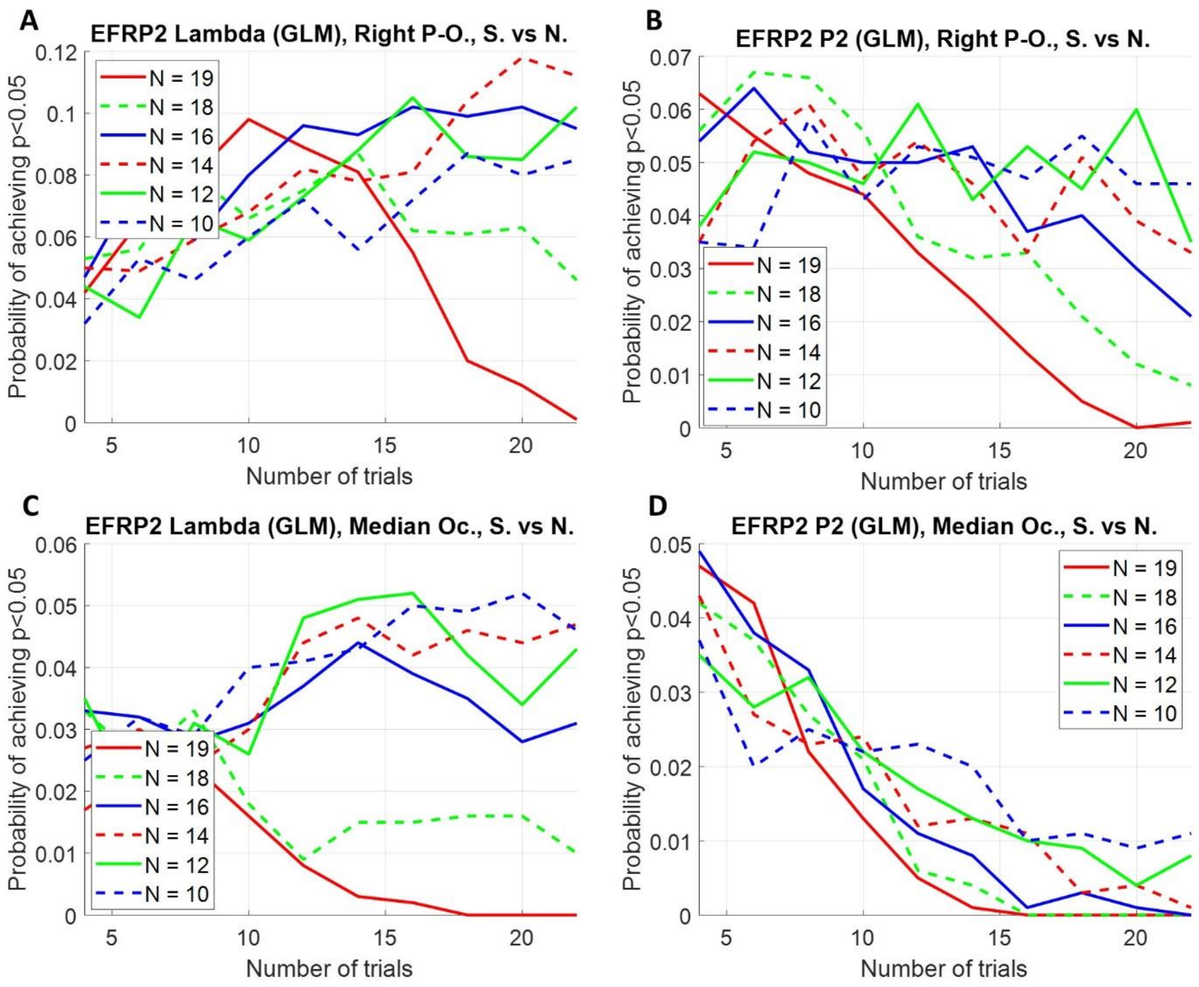

Supplementary Figure 5: Probability for achieving the significant difference on Lambda response (A, C) and the P2 component (B, D) of the second and subsequent EFRP, between surprise and neutral conditions, at the right parieto-occipital site (A, B) and at the median occipital site (C, D)
