# Supplementary figures and images for: Temporal Dynamics of Natural Static Emotional Facial Expressions Decoding: A Study Using Event- and Eye Fixation-Related Potentials
Source: Front Psychol. 2018 Jul 12;9:1190. doi: 10.3389/fpsyg.2018.01190 (PMC6052106; doi:10.3389/fpsyg.2018.01190)

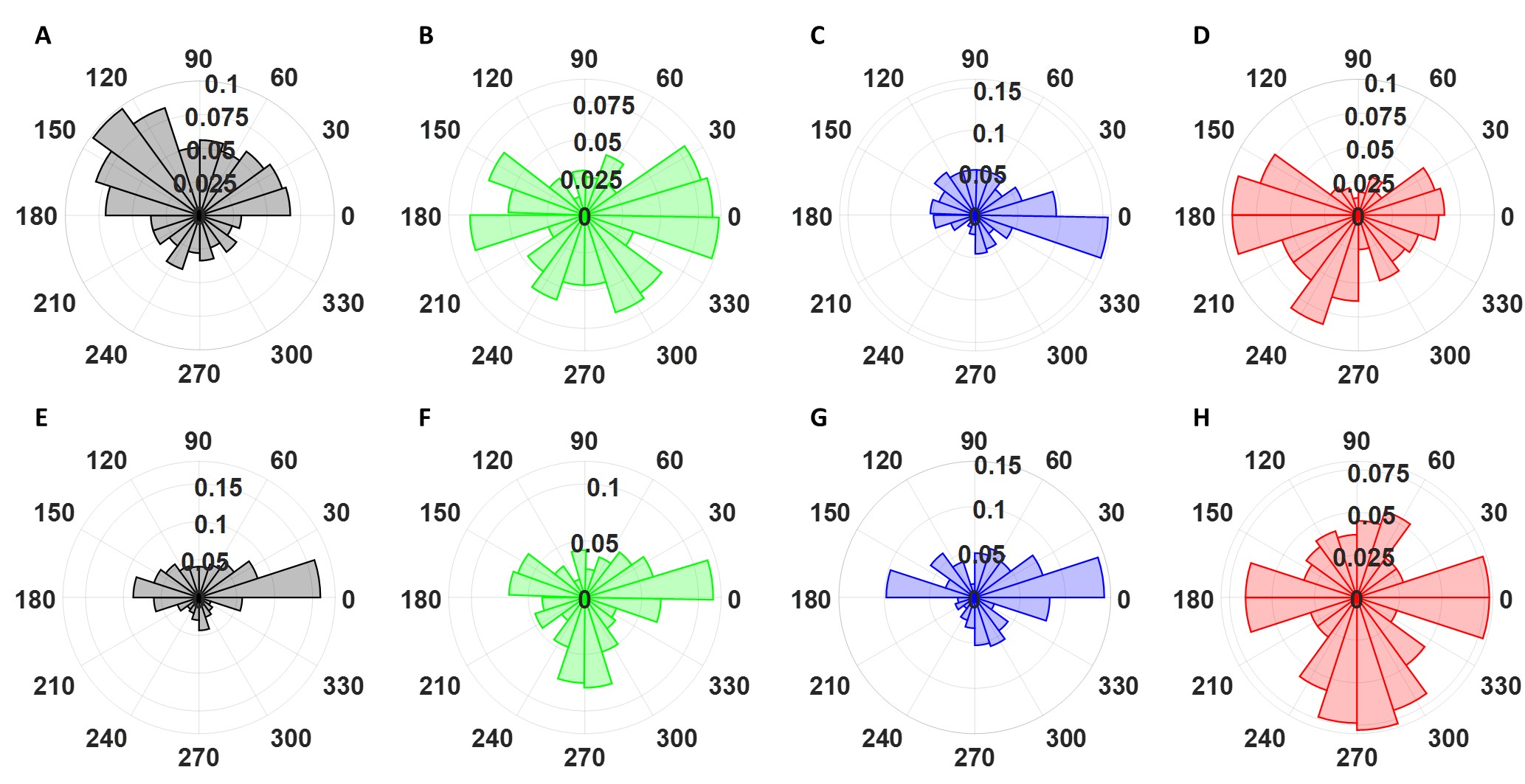

Supplement: Supplementary file 2 [file Image_1.JPEG]

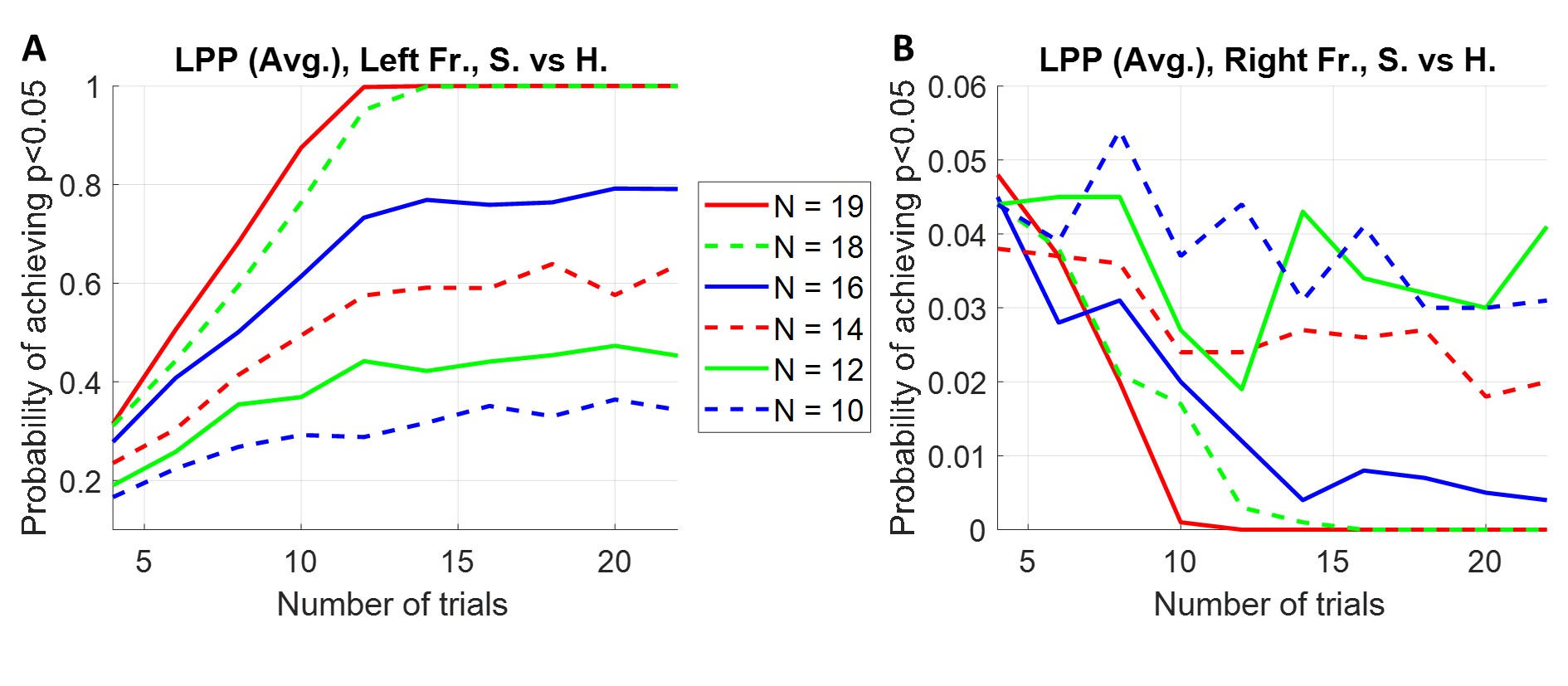

Supplement: Supplementary file 3 [file Image_2.JPEG]

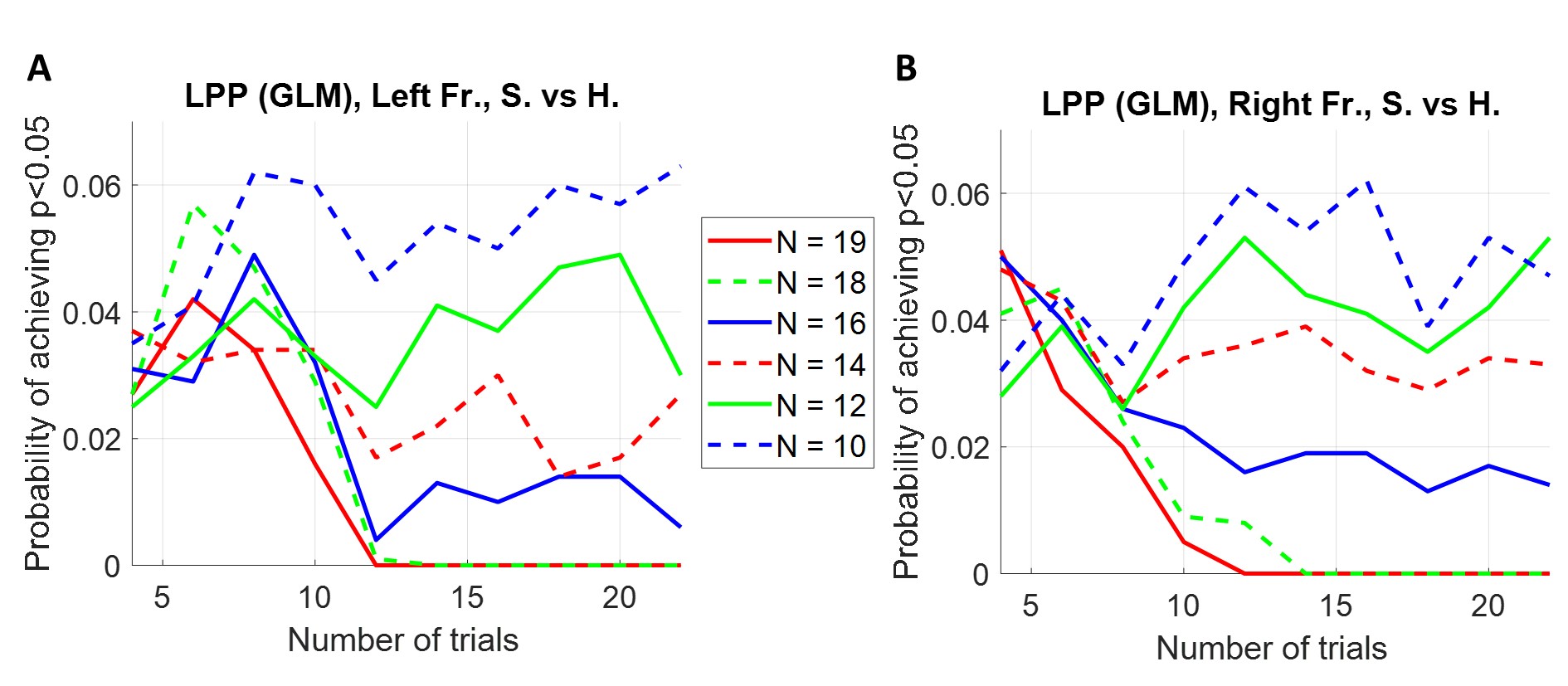

Supplement: Supplementary file 4 [file Image_3.JPEG]

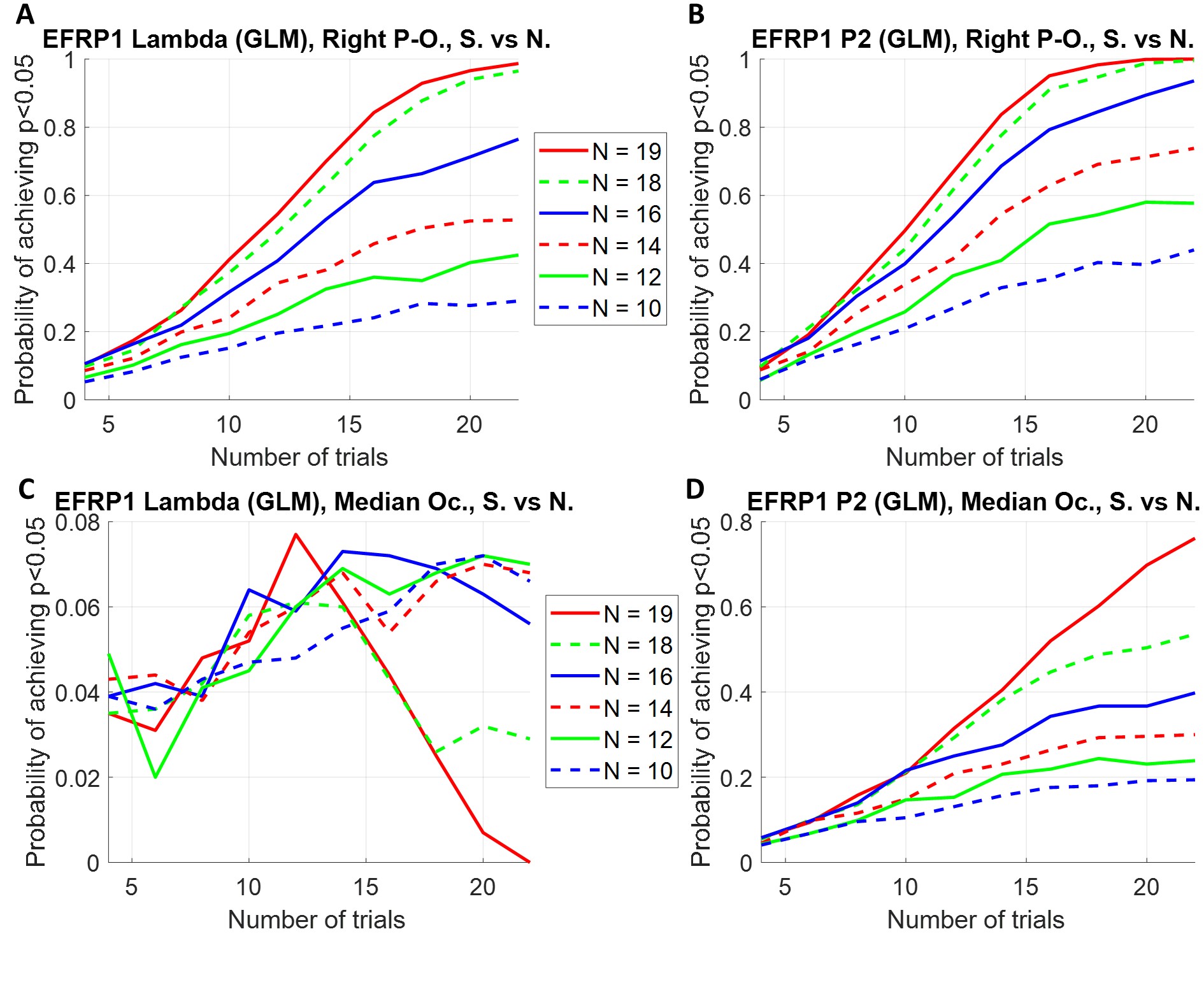

Supplement: Supplementary file 5 [file Image_4.JPEG]

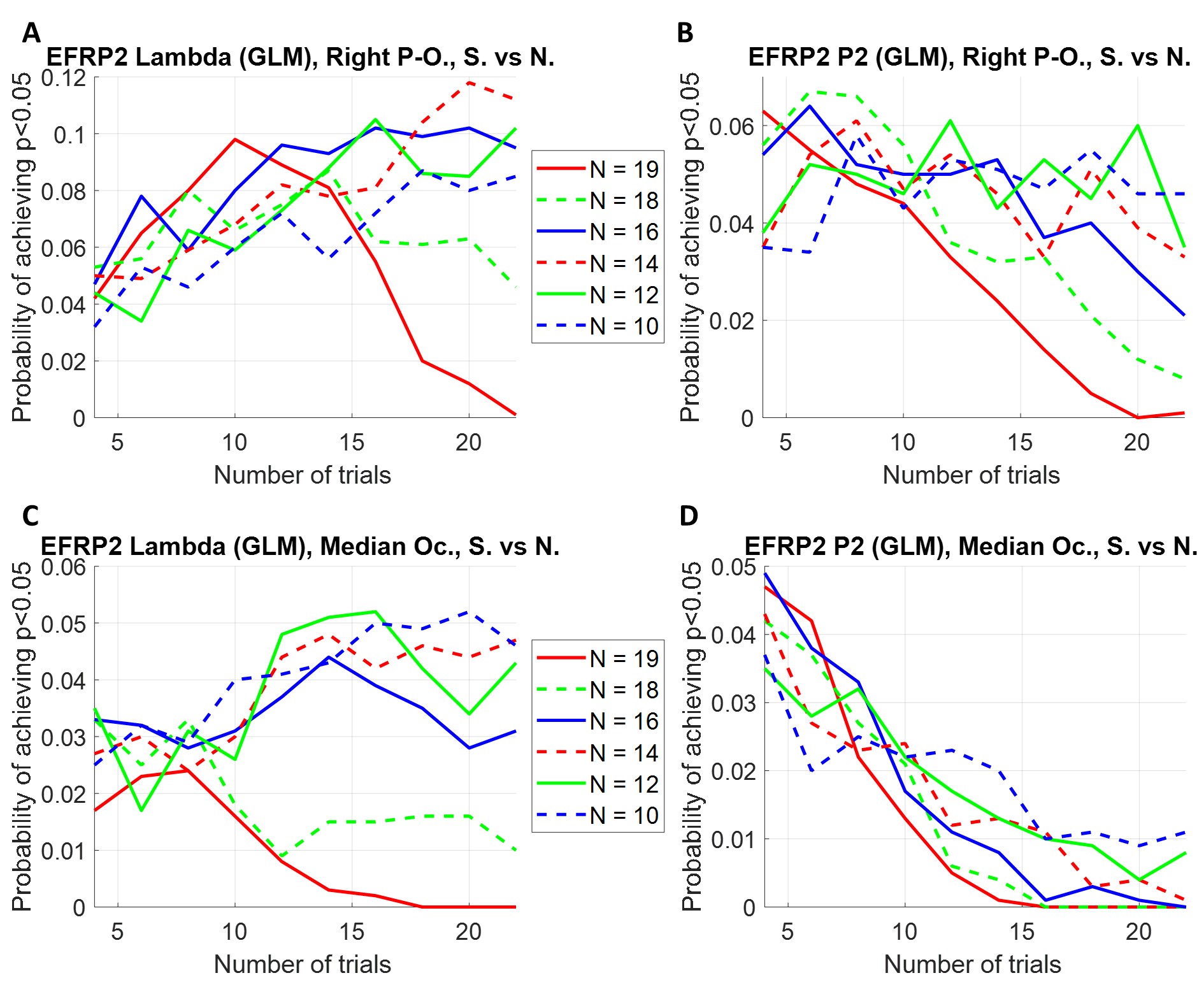

Supplement: Supplementary file 6 [file Image_5.JPEG]
